# Effect of an E-mental Health Approach to Workers' Health Surveillance versus Control Group on Work Functioning of Hospital Employees: A Cluster-RCT

**DOI:** 10.1371/journal.pone.0072546

**Published:** 2013-09-12

**Authors:** Sarah M. Ketelaar, Karen Nieuwenhuijsen, Fania R. Gärtner, Linda Bolier, Odile Smeets, Judith K. Sluiter

**Affiliations:** 1 Coronel Institute of Occupational Health, Academic Medical Center, University of Amsterdam, Amsterdam, The Netherlands; 2 Innovation Center of Mental Health and Technology (I.COM), Trimbos Institute, Netherlands Institute of Mental Health and Addiction, Utrecht, The Netherlands; Maastricht University Medical Centre, The Netherlands

## Abstract

**Objective:**

To evaluate an e-mental health (EMH) approach to workers' health surveillance (WHS) targeting work functioning (WF) and mental health (MH) of healthcare professionals in a randomised controlled trial.

**Methods:**

Nurses and allied health professionals (N = 1140) were cluster-randomised at ward level to the intervention (IG) or control group (CG). The intervention consisted of two parts: (a) online screening and personalised feedback on impaired WF and MH, followed by (b) a tailored offer of self-help EMH interventions. CG received none of these parts. Primary outcome was impaired WF (Nurses Work Functioning Questionnaire), assessed at baseline and after three and six months. Analyses were performed in the positively screened subgroup (i) and in all participants (ii).

**Results:**

Participation rate at baseline was 32% (N_IG_ = 178; N_CG_ = 188). Eighty-two percent screened positive for at least mild impairments in WF and/or MH (N_IG_ = 139; N_CG_ = 161). All IG-participants (N = 178) received part (a) of the intervention, nine participants (all positively screened, 6%) followed an EMH intervention to at least some extent. Regarding the subgroup of positively screened participants (i), both IG and CG improved over time regarding WF (non-significant between-group difference). After six months, 36% of positively screened IG-participants (18/50) had a relevant WF improvement compared to baseline, versus 28% (32/115) of positively screened CG-participants (non-significant difference). In the complete sample (ii), IG and CG improved over time but IG further improved between three and six months while CG did not (significant interaction effect).

**Conclusions:**

In our study with a full compliance rate of 6% and substantial drop-out leading to a small and underpowered sample, we could not demonstrate that an EMH-approach to WHS is more effective to improve WF and MH than a control group. The effect found in the complete sample of participants is not easily interpreted. Reported results may be useful for future meta-analytic work.

**Trial Registration:**

Dutch Trial Register NTR2786

## Introduction

Nurses have a high risk of developing common mental health complaints, such as distress, depression, and anxiety [1]–[3]. Impaired mental health of employees in healthcare occupations can have serious adverse effects, endangering the health and safety of not only themselves but also their patients. A study by Gärtner and colleagues found that impaired mental health in nurses and allied health professionals affects several aspects of their work functioning, including cognitive aspects (e.g. staying alert) and causing incidents at work [4]. Another study by Letvak and colleagues showed that depression in nurses was associated with presenteeism, which is in turn associated with patient falls, medication errors, and lower self-reported quality of care [5]. Adding to this, increased levels of psychological distress, even in a mild form, have been found to be associated with an increased likelihood of obtaining a disability pension in later life [6]. To sustain nurses' and allied health professionals' mental health and to enable them to remain healthy and well-functioning in their profession until retirement age, it is crucial to periodically screen these employees and provide interventions to improve their mental health and work functioning.

A potentially promising method for the early detection of impaired mental health and subsequent treatment in nurses and allied health professionals, is offering a mental module for workers' health surveillance (WHS). Although attention has been paid to the occupational hazards of healthcare employees [7], WHS targeting work functioning and mental health of nurses and allied health professionals has, to our knowledge, not been reported before.

WHS is an important component of occupational healthcare [8]. It is a means to implement preventive action by identifying and treating health complaints relevant to work, and it should be an essential component of programmes aimed at the protection of employees [9]. In the Netherlands, it has three aims: 1) to prevent the onset, recurrence, or worsening of work-related diseases, 2) to monitor and promote work-related health, and 3) to monitor and improve work functioning and employability [10]. It can be used to periodically monitor employees' health and work functioning to detect impairments early and to bring timely interventions into action to prevent further impairment. It is recommended to apply a job-specific assessment, to allow for tailoring of interventions to the specific detected work functioning impairments as fitting as possible [11]. In this study, we detect early signs of impaired mental health and impaired work functioning in nurses and allied health professionals, and offer interventions using an e-mental health approach.

E-mental health (EMH) is the use of information and communication technology, and in particular the many technologies related to the Internet, to support and improve mental health [12]. Applying EMH might be a useful and feasible approach to perform a mental module for WHS. Online screening is a practical and efficient method to screen for self-reported impaired work functioning and impaired mental health. Furthermore, EMH offers possibilities regarding the subsequent interventions. Ritterband and colleagues defined Internet interventions as typically focused on behavioral issues, aiming to institute behavior change and subsequent symptom improvement, usually self-paced, interactive, and tailored to the user, and making use of the multimedia format offered by the Internet [13]. EMH interventions exist which target a wide variety of common mental disorders such as depression, anxiety, panic, phobias, and various addictions. Unguided self-help EMH interventions have been found to have positive outcomes for a variety of mental health aspects (e.g. Warmerdam et al. [14]; Farrer et al. [15]; Riper et al. [16]; Blankers et al. [17]; Billings et al. [18]), although to our knowledge their effects on work functioning have not been studied in a specific working population such as nurses and allied health professionals. Moreover, EMH interventions have thus far only been offered as stand-alone interventions for a specific mental health complaint. In our study, we offer a choice of EMH interventions, tailored to the specific complaints as indicated by the individual's screening results.

In this paper, we study the effect of an EMH-approach to WHS targeting work functioning and mental health of hospital-employed nurses and allied health professionals, on their work functioning, distress, work-related fatigue, posttraumatic stress, and work ability in a cluster-randomized controlled trial. We hypothesized that WHS, consisting of online screening on impaired work functioning and impaired mental health followed by personalised feedback and a tailored offer of self-help EMH interventions, will improve work functioning and mental health.

## Methods

The protocol for this trial and supporting CONSORT checklist are available as supporting information; see Checklist S1 and Protocol S1 (http://www.biomedcentral.com/1471-2458/11/290).

### Ethics statement

The Medical Ethics Committee of the Academic Medical Center Amsterdam approved this study (for approved protocol see Protocol S2). All participants gave their written informed consent before taking part.

### Study design

The study was designed as a cluster-randomised trial with block randomisation carried out at ward level. In order to guarantee allocation concealment, randomisation was performed by one researcher (KN) who was not involved in the practical recruitment of employees, using the computer software program Nquery Advisor with a block size of three. The complete trial included two intervention groups and one control group [19]. The present study compared one of the intervention groups, the e-mental health approach (EMH-approach) group, to the control group. The other intervention group consisted of an invitation for a preventive consultation with an occupational physician. A pre-randomisation procedure with incomplete-double-consent design was applied [20], meaning that individuals were only informed about their own group.

Outcome measures were obtained from all participants at baseline (March 2011) and follow-up measures were obtained three and six months after baseline.

The design, conduct and reporting of this study adhere to the Consolidated Standards of Reporting Trials guidelines [21], [22]. Details of the study design are reported elsewhere [19]. The trial registration number of the study is NTR2786 (Dutch Trial Register: http://www.trialregister.nl).

### Participants

The study population of the complete trial was formed by all nurses, including surgical nurses and anaesthetic nurses, and allied health professionals (such as physiotherapists and radiotherapists) employed at one academic hospital in the Netherlands (N = 1731). Nurses and allied health professionals form two large groups of hospital employees, and many of their work demands and work conditions are similar. Since it regarded a preventive study, participants were included if they were not, or were not expecting to be on sick leave for more than two weeks at baseline.

**Figure 1:**
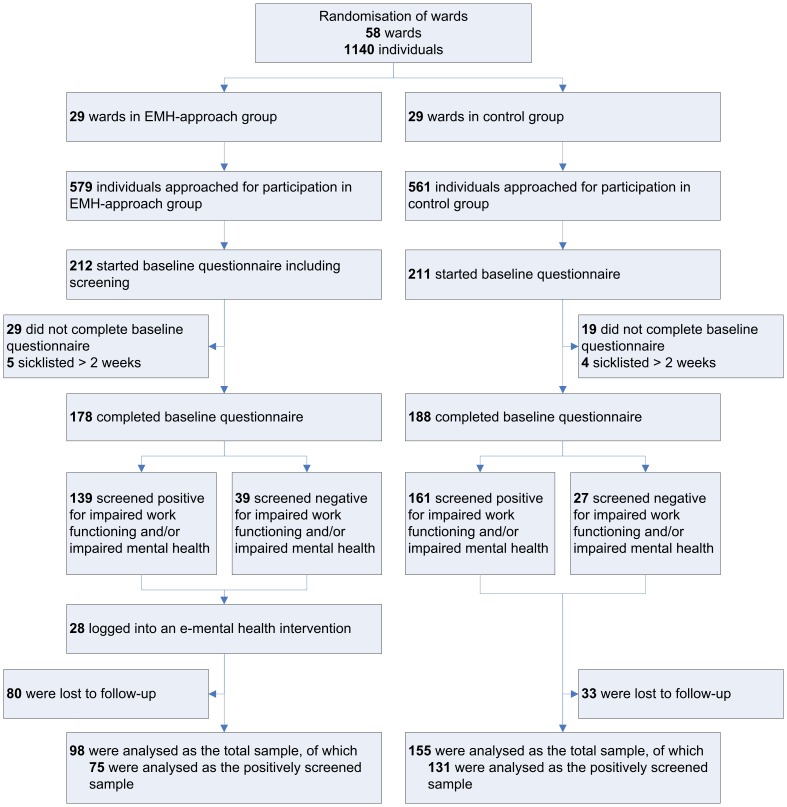
Flow of participants through the trial.

All eligible employees were invited for participation in the study. To detect a clinically significant effect (effect size≥.33), while conducting the tests with alpha = .05 (two-tailed) and power = .80, and allowing for possible cluster effects and loss to-follow-up, the minimum required sample size was 718 participants for the complete trial [19]. After randomization at ward level (N = 86), 29 wards with 579 employees were assigned to the EMH-approach group and 29 wards with 561 employees to the control group (Figure 1).

### Procedure

In March 2011, potential participants received an invitation by e-mail to fill out the online baseline questionnaire which could be filled out at any time during six weeks. It was possible to discontinue the questionnaire and complete it after logging in again. Three reminders were sent, as well as an information letter to their home address. Those who had completed the baseline questionnaire were invited to fill out the follow-up questionnaires three and six months after baseline.

### Intervention

#### E-mental health approach group

At baseline, participants in the EMH-approach group were screened on the following aspects (for details see Table 1 and Gärtner and colleagues [19]): impaired work functioning, distress, work-related fatigue, risky drinking behaviour, depression including suicide risk, anxiety, panic disorder, and posttraumatic stress. Participants received personalised feedback on their screening results immediately after filling out the baseline questionnaire, both onscreen and in an e-mail.

**Table 1:** Screening instruments and cut-off points.

Aspect	Instrument	Cut-off point
Impaired work functioning	Nurses Work Functioning Questionnaire (7 subscales) [4]	Red score on ≥1 subscales and/or orange score on ≥3 subscales [19]
Distress	Four-Dimensional Symptoms Questionnaire, distress subscale [32], [33]	Total score≥11 [34]
Work-related fatigue	Need for recovery subscale of the Dutch Questionnaire on the Experience and Evaluation of Work [35]	Standardised total score≥54.5 [36]
Risky drinking behaviour	AUDIT-C [49]	Total score≥5 for men, ≥4 for women [50]
Depression	Brief Symptom Inventory, depression subscale [51]	Mean score≥0.42 [52]
(Suicide risk)	(One item from Brief Symptom Inventory, depression subscale [51])	(Score≥3 on 0–4 scale)
Anxiety	Brief Symptom Inventory, anxiety subscale [51]	Mean score≥0.42 [52]
Panic disorder	Patient Health Questionnaire [53], only assessed for participants identified as having anxiety complaints	2 answers affirmative on the first 4 items plus 4 symptoms affirmative on the following 11 items [54]
Posttraumatic stress	Dutch translation of the Impact of Event Scale [37], [38]	Total score≥26 [39]

The personalised feedback was followed by an invitation for a tailored offer of self-help EMH interventions, on the basis of an algorithm based on the specific symptoms and the work-relatedness of the symptoms (available as supporting information, see Algorithm S1). Participants were mostly offered a choice of two or three EMH interventions to leave room for personal preferences. Participants who screened negative on all mental health complaints were invited to follow an EMH intervention aimed at enhancing and retaining their mental fitness.

The EMH interventions used in this study are self-help interventions on the Internet aimed at reducing specific mental health complaints or enhancing wellbeing. The interventions are mainly based on the principles of cognitive behavioural therapy and combine a variety of aspects, e.g. providing information and advice, weekly assignments, the option of keeping a diary and a forum to get in contact with others who have similar complaints. The EMH interventions were developed as stand-alone interventions by the Trimbos Institute (Netherlands Institute of Mental Health and Addiction) at an earlier stage. The following EMH interventions were used in the study:

*Psyfit* [*23*] *:* aimed at enhancing mental fitness. Also applied for healthy participants.
*Strong at work* [*24*] *:* aimed at gaining insight into work stress and learning skills to cope with it.
*Colour your Life* [*25*] *:* aimed at tackling depressive symptoms.
*Don't Panic Online* [*26*] *:* aimed at reducing panic symptoms for subclinical and mild cases of panic disorder.
*Drinking Less* [*16*] *:* aimed at reducing risky drinking behaviour.

*Psyfit* was found to be effective in decreasing symptoms of depression and anxiety and improving well-being and vitality [27]. Warmerdam and colleagues [14] showed that *Colour your Life* resulted in significantly lower depression and anxiety scores compared to a waiting-list control group and to significantly higher quality of life scores. The number of participants showing clinically relevant change regarding depression after 12 weeks was significantly higher. Spek and colleagues also found a significantly larger improvement in depressive symptoms compared to a waiting-list control group [28], [29]. *Drinking Less* resulted in more participants who reduced their drinking successfully to within guideline norms, and a significantly larger decrease in mean weekly alcohol consumption than a control group [16].

In case of positive screening on impaired work functioning (regardless of their mental health status), participants received an onscreen educational leaflet on how to improve their work functioning (available upon request).

#### Control group

Participants in the control group filled out the same baseline questionnaire as the EMH-approach group, but did not receive an intervention, and thus no screening results either. However, they were informed that they would receive personalised feedback and a tailored offer of self-help EMH interventions after six months, following the six months follow-up questionnaire.

### Measures

All outcomes were measured at baseline and at three and six months follow-up.

#### Primary outcome

The primary outcome of this study was impaired work functioning, measured with the total score of the Nurses Work Functioning Questionnaire (NWFQ) [4]. This questionnaire has been developed to assess impaired work functioning in nurses and allied health professionals. In the screening phase, all seven of the original subscales were used. Participants scored either green, orange or red on each subscale. A red score on one or more subscales and/or three or more orange scores led to case identification of impaired work functioning (i.e. scoring above cut-off point on impaired work functioning) [19].

Only six of the seven original NWFQ subscales were used for the outcome measure, in contrast to what was described in the trial's design study [19], because the reproducibility of the *impaired decision-making* subscale was found to be poor [30]. The total score on the NWFQ was calculated with the 47 items of the remaining six subscales, with a total score range of 0–100, a higher score indicating more severely impaired work functioning.

The difference between the EMH-approach group and the control group regarding impaired work functioning was investigated using the continuous outcome and the percentage of individuals who had improved relevantly at follow-up [31].

#### Secondary outcomes

The secondary outcomes included distress, work-related fatigue, posttraumatic stress, and work ability.

Distress was measured with the *distress* subscale of the Four-Dimensional Symptoms Questionnaire (4DSQ) [32], [33]. The 16-item questionnaire uses a 5-point response scale (0 = *no*, 4 = *very often*) and has a total score range of 0–32, a higher score indicating a higher level of distress (cut-off point ≥11 [34]).

Work-related fatigue after working time was measured using the *need for recovery* subscale of the Dutch Questionnaire on the Experience and Evaluation of Work (QEEW) [35]. The 11-item questionnaire with dichotomous response categories (*yes, no*) has a total score range of 0–11 and a standardized score range of 0–100, a higher score indicating a higher level of work-related fatigue (cut-off point ≥54.5 [36]).

Posttraumatic stress was measured with the Dutch version of the Impact of Event Scale [37], [38]. The 15 items can be answered on a 4-point response scale (0 = *not at all*, 3 = *often*). Total scores range from 0–75, a higher score indicating a higher level of posttraumatic stress (cut-off point ≥26 [39]).

Work ability was assessed with the first item of the Work Ability Index (WAI) [40]. This item concerns the evaluation of current work ability compared to their lifetime best on an 11-point scale (0 = *completely unable to work*, 10 = *work ability at its best*), a higher score indicating a higher level of work ability.

### Statistical analyses

All participants who completed the baseline questionnaire and who screened positive on impaired work functioning and/or impaired mental health (the targeted sample) were analysed, as the work functioning and mental health of these participants could be expected to change due to the intervention. However, since this was not pre-specified in the trial registration, the analyses were also performed with the total sample of participants (i.e. all participants, regardless of their screening results).

To describe participants, we used the following demographics: sex, age, occupation, specialization (yes/no), years of working experience, working hours per week, and type of contract. Additionally, the number of participants scoring above cut-off point for impaired work functioning and mental health complaints were calculated.

The analyses were performed at the level of the individual employee, according to the intention-to-treat principle. The significance level was set at α = .05. All analyses were carried out using the statistical package IBM SPSS Statistics 19.

#### Drop-out analysis

A drop-out analysis was performed to detect whether dropping out of the trial was related to the primary outcome impaired work functioning, and to identify potential predictive variables of drop-out. Dropping out of the trial was defined as completing the baseline and three months follow-up questionnaires, but not the six months follow-up questionnaire; or completing the baseline questionnaire, but none of the follow-up questionnaires. Differences between drop-outs and non drop-outs in impaired work functioning over time in both separate groups were explored in graphs. If different patterns of the effect after three months were detected, a Mann-Whitney U test was performed to test the significance of the differences. In the event of statistically significant differences, a multiple logistic regression analysis was performed with drop-out as the dependent variable, to identify potential predictive variables for drop-out. Screening positive on mental health complaints at baseline (yes/no) and age were included as the independent variables, as we expected that these two aspects might be related to dropping out of the trial. If the multiple logistic regression analysis showed one or both of these aspects to have a statistically significant effect on drop-out, they were included as a covariate in the effect analyses [41].

#### Effect analysis

To analyse the differences over time between the EMH-approach group and the control group on each outcome, Linear Mixed Models (LMM) were applied. If the assumption of a normal distribution of residuals was not met, a log-transformation was used for the LMM and the median and range were used to describe the outcome. Otherwise, the mean and standard deviation were used to describe the outcome.

For each outcome, the scores at three and six months follow-up were included as dependent variables in the LMM, while the baseline score was included as a covariate. The main effects of *group* and *time of measurement*, and the interaction of group*time of measurement were included as fixed effects in the model. *Ward* (the cluster level) and *subject* (the individual level) were included as random effects; however if the cluster level did not have a statistically significant effect, it was considered negligible and was therefore excluded from the model. The effects of interest were the main effect of *group* (interpreted as the difference between the groups from baseline to six months follow-up) and the interaction effect of group*time of measurement (interpreted as the difference between the groups from three to six months follow-up).

For all outcomes in the positively screened subgroup, we calculated Cohen's d [42] by determining the mean difference between the baseline score and the score at follow-up, divided by the pooled standard deviation. For Cohen's d, a score of 0.2 to 0.5 can be considered a small effect, 0.5 to 0.8 a medium effect, and greater than 0.8 a large effect [42].

Additionally, the relative change scores of individuals on impaired work functioning after three and after six months of follow-up compared to their baseline score were calculated. Individuals with a relative improvement on their NWFQ total score of 40% or more, which is the minimal important change (MIC) value of the NWFQ total scale [31], were defined as *relevantly improved*. The percentages of individuals who had improved relevantly in each group were compared using a Fisher's exact test, for both three months and six months follow-up.

## Results

### Participant flow

Figure 1 presents the flow of participants through the trial. From March 15^th^ until April 26^th^, 423 employees (37%) started on the baseline questionnaire. Of those, 366 (32% of invited employees) were eligible for participation, 178 (31%) in the EMH-approach group and 188 (34%) in the control group. In the EMH-approach group, 80 participants (45%) were lost to follow-up, compared to 33 participants (18%) in the control group. Reasons for withdrawal were not assessed. Fifty-six participants (31%) in the EMH-approach group and 126 participants (67%) in the control group completed all three questionnaires.

Analyses were performed on the participants who screened positive (primary outcome: EMH N = 75, 54%; control N = 131, 81%), and additionally on all participants (primary outcome: EMH N = 98, 55%; control N = 155, 82%) who had participated in at least one follow-up.

Twenty-two participants (17 positively screened) logged into *Psyfit*, seven logged into *Strong at work*, four logged into *Colour your Life*, and no-one logged into *Don't Panic Online* or *Drinking Less*. Nine participants (all positively screened) followed an intervention to at least some extent (*Psyfit*: 6, *Colour your Life*: 3).

### Study population at baseline

As shown in Table 2, the study groups were quite similar regarding demographic and occupational characteristics. The majority of participants were female and employed as a nurse. Participants in the EMH-approach group had a younger average age of 37, compared to 42 in the control group. The participants worked an average of 31 hours per week and most of them had a permanent position in the hospital. Around 4/5^th^ of participants screened positive on work functioning impairments and/or impaired mental health, more participants in the control group (N = 161, 86%) than in the EMH-approach group (N = 139, 78%).

**Table 2:** Participant characteristics at baseline for the EMH-approach group and the control group.

	Total sample				Positively screened sample			
	EMH-approach (N = 178)		Control (N = 188)		EMH-approach (N = 139)		Control (N = 161)	
Variable	n	(%)	n	(%)	n	(%)	n	(%)
*Sex*								
Female	147	(83)	145	(77)	113	(81)	126	(78)
*Age in years (mean (SD))*	37	(12)	42	(11)	38	(12)	42	(12)
*Occupation*								
Nurse	129	(73)	134	(71)	99	(71)	115	(71)
Nurse practitioner	11	(6)	22	(12)	7	(5)	18	(11)
Surgical nurse	0	(0)	5	(3)	0	(0)	5	(3)
Anesthetic nurse	0	(0)	0	(0)	0	(0)	0	(0)
Allied health professional	38	(21)	27	(14)	33	(24)	23	(14)
*Nursing specialization*								
Yes	74	(57)	86	(64)	57	(58)	75	(65)
*Years of experience (mean (SD))*	10	(10)	11	(10)	11	(10)	11	(10)
*Working hours per week according to contract (mean (SD))*	31	(5)	31	(6)	31	(5)	31	(6)
*Type of contract*								
Permanent position	160	(91)	174	(93)	125	(91)	150	(94)
Fixed-term contract	13	(7)	12	(6)	11	(8)	9	(6)
Temporary employment	0	(0)	0	(0)	0	(0)	0	(0)
Other	3	(2)	1	(1)	1	(1)	1	(1)
*Impaired work functioning* * *(above cut-off)*								
Work functioning impairments	107	(60)	131	(70)	107	(77)	131	(81)
*Impaired mental health (above cut-off)*								
Impaired mental health (above cut-off of one or more of the six mental health aspects)	109	(61)	119	(63)	109	(78)	119	(74)
Distress	41	(23)	48	(26)	41	(30)	48	(30)
Work related fatigue	61	(34)	65	(35)	61	(44)	65	(40)
Post traumatic stress	24	(14)	19	(10)	24	(17)	19	(12)
*Screened positive on impaired work functioning and/or impaired mental health*	139	(78)	161	(86)	139	(100)	161	(100)

* Note: Work functioning is presented here including the subscale *impaired decision-making*, as it was included in the screening at baseline.

### Drop-out analysis

Graphs in which the scores of drop-outs and non drop-outs on the primary outcome were compared, showed that in both groups drop-outs had a worse score on impaired work functioning (EMH baseline median = 13, 3 mn follow-up median = 12; C baseline median = 14, 3 mn follow-up median = 11) than non drop-outs (EMH baseline median = 9, 3 mn follow-up median = 8; C baseline median = 12, 3 mn follow-up median = 8) at baseline and three months follow-up. A Mann-Whitney U test identified that these differences were statistically significant in the EMH-approach group (baseline U = 4.688, p = .01; 3 mn follow-up U = 970, p = .04) and in the entire group of participants (baseline U = 19.202,5, p = .01; 3 mn follow-up U = 5.079,5, p = .01). In a subsequent logistic regression analysis, age was identified as a statistically significant predictor of drop-out in the entire group (p = .02, younger participants had a higher chance of drop-out), but screening positive for mental health complaints at baseline (yes/no) was not (p = .16). Therefore, age was included as a covariate in the effect analyses.

### Intervention effects

The results in Table 3 refer to the group of participants who screened positive for impaired work functioning and/or mental health impairments at baseline. The relative frequency of participants who scored above cut-off point on the outcome measures and the mean and median scores (in case of a non-normal distribution) are presented for baseline and both follow-up points, as well as the results of the LMM analyses.

**Table 3:** Descriptives and analysis results on primary and secondary outcomes of the positively screened sample at baseline, 3 and 6 months follow-up.

		E-mental health approach group				Control group				p-value (LMM analyses)**	
		Relative frequency above cut-off (%)	Median (range)	Mean (SD)	Effect size (95% CI)	Relative frequency above cut-off (%)	Median (range)	Mean (SD)	Effect size(95% CI)	group	group*time
**Primary outcome**											
*Impaired work functioning*	b	91/139 (66)	14 (0–56)			110/161 (68)	14 (0–54)			.771	.283
*(NWFQ 0–100)* *	3 mn	33/62 (53)	10 (0–39)		.19 (−.16–.55)	61/124 (49)	10 (0–38)		.26 (.01–.51)		
	6 mn	19/52 (37)	8 (0–41)		.16 (−.22–.55)	60/116 (52)	10 (0–44)		.24 (−.01–.50)		
**Secondary outcomes**											
*Distress*	b	41/139 (30)	7 (0–32)			48/161 (30)	6 (0–32)			.592	.828
*(4DSQ, 0–32)*	3 mn	9/61 (15)	4 (0–29)		.20 (−.16–.56)	25/123 (20)	5 (0–29)		.26 (.01–.51)		
	6 mn	10/52 (19)	5 (0–29)		.07 (−.32–.45)	26/116 (22)	5 (0–30)		.14 (−.11–.40)		
*Work-related fatigue*	b	61/139 (44)		44 (28)		65/161 (40)		39 (30)		.617	.732
*(QEEW, 0–100)*	3 mn	22/61 (36)		36 (31)	.16 (−.20–.52)	42/123 (34)		35 (30)	.12 (−.13–.37)		
	6 mn	14/52 (27)		34 (30)	.02 (−.36–.41)	39/116 (34)		37 (31)	.02 (−.24–.27)		
*Posttraumatic stress*	b	24/139 (17)	3 (0–71)			19/161 (12)	3 (0–48)			.357	.124
*(IES, 0–75)*	3 mn	10/61 (16)	1 (0–48)		.07 (−.29–.42)	13/122 (11)	0 (0–62)		.31 (.05–.56)		
	6 mn	5/51 (10)	0 (0–31)		.24 (−.15–.63)	9/116 (8)	0 (0–48)		.26 (.00–.51)		
*Work ability*	b			7 (1)				8 (2)		.483	.552
*(WAI, 0–10)*	3 mn			8 (1)	.14 (−.21–.50)			8 (1)	−.01 (−.26–.25)		
	6 mn			8 (2)	.05 (−.34–.44)			8 (1)	.01 (−.25–.26)		

* Note: NWFQ total scores were calculated without the subscale *impaired decision-making*.

** Number analysed in EMH-approach group: N = 75 (impaired work functioning), N = 74 (distress and work-related fatigue), N = 73 (posttraumatic stress and work ability); numbers analysed in Control group: N = 131 (all outcomes).

b, baseline; 3 mn, follow-up after 3 months; 6 mn, follow-up after 6 months.

Since the random effect of ward (the cluster level) was not statistically significant in any of the analyses, it was excluded from the model in the LMM analyses.

#### Impaired work functioning (primary outcome)

The EMH-approach group and the control group improved to a similar degree between baseline and three months follow-up. The EMH-approach group improved further between three and six months, while the control group remained at approximately the same level. As shown in Table 3, in the LMM analysis of impaired work functioning in the positively screened sample of participants, no statistically significant difference between the EMH-approach group and the control group was identified (main effect of group p = .77; interaction effect of group*time p = .28). The effect size estimate after three and six months was comparably low in both groups.

In the LMM analysis of the total sample of participants (data not shown in table), no significant effect of group was found (p = .68), but a significant interaction effect of group*time was found (p = .04), suggesting there to be a different pattern of scores on impaired work functioning from three to six months follow-up between the EMH-approach group and the control group. A closer look at the median scores on impaired work functioning revealed that both groups improved to a similar degree between baseline and three months follow-up, and that the EMH-approach group further improved between three and six months follow-up while the control group slightly deteriorated.

In Table 4, the percentages of individual employees with a relevant improvement on work functioning after three and after six months compared to their baseline score are shown. After three months, in the positively screened sample as well as the total sample, roughly the same percentage of participants in both groups had improved relevantly regarding work functioning compared to their baseline score. After six months, more participants in the EMH-approach group than in the control group had improved relevantly compared to baseline, in both the positively screened sample (EMH 36%; control 28%) and the total sample (EMH 40%; control 30%). However, these differences were not statistically significant (p = .36 and p = .21, respectively).

**Table 4:** Participants whose work functioning had improved with at least the minimal important change at 3 and 6 months follow-up compared to baseline: descriptives and analysis results.

		EMH-approach	Control group	p-value
		Relative frequency (%)	Relative frequency (%)	(Fisher's exact test)
Positively screened sample	3 mn	18/60 (30%)	37/123 (30%)	1.000
	6 mn	18/50 (36%)	32/115 (28%)	.357
Total sample	3 mn	24/80 (30%)	46/142 (32%)	.765
	6 mn	27/68 (40%)	40/134 (30%)	.206

3 mn, follow-up after 3 months; 6 mn, follow-up after 6 months.

#### Secondary outcomes

As shown in Table 3, both groups improved over time regarding distress, work-related fatigue, and posttraumatic stress, with the largest improvement between baseline and three months of follow-up. On distress and work-related fatigue, the EMH-approach group had a larger overall improvement than the control group (non-significant). In the LMM analyses on distress, work-related fatigue, posttraumatic stress, and work ability in the positively screened sample, no statistically significant differences were found between the EMH-approach group and the control group (main effect of group .36≤p≤.62; interaction effect of group*time .12≤p≤.83). Effect sizes in both groups were fairly similar, small to non-relevant.

In the LMM analyses on the secondary outcomes in the total sample of participants (data not shown in table), no significant differences were found between the EMH-approach group and the control group either (.31≤p≤.97).

## Discussion

The results of our study suggest that an e-mental health (EMH) approach of workers' health surveillance (WHS), consisting of online screening on impaired work functioning and impaired mental health followed by personalised feedback and a tailored offer of self-help EMH interventions, shows no significant improvement in impaired work functioning, distress, work-related fatigue, posttraumatic stress, and work ability to a larger extent than a control group. Compliance to the EMH interventions was low, which impedes drawing a conclusion about the effect of this part of the intervention. Screening and personalised feedback was received by all participants in the intervention group. Although the study had insufficient power, the low effect sizes do not give reason to expect a relevant effect of screening and feedback. The outcomes may be of value for future meta-analytic work.

One third of the employees who were invited, participated in the study. Of these participants, more than 80% screened positive for at least mild impairments in work functioning and/or mental health. Both the intervention group and the control group improved over time on work functioning, distress, work-related fatigue, and posttraumatic stress, with no statistically significant difference between the groups. However, when including all participants in the analyses and not only those who had screened positive on impairments at baseline, the work functioning of the EMH-approach group showed a significantly different pattern compared to the control group, as the EMH-approach group further improved between three and six months after baseline while the control group did not. After six months, a relevant improvement of work functioning was found for 36% of positively screened participants in the intervention group and 28% in the control group, but the difference between the groups was non-significant.

### Interpretation of results

First of all, our study had a high percentage of participants who screened positive for at least mild impairments. This included screening positive on impairments in work functioning, on one or more mental health complaints or both. In choosing our cut-off points, we aimed for high sensitivity, since we did not want to miss participants who might need help. The cut-off points that we applied for the mental health complaints were all validated. However, high sensitivity generally comes at the expense of high specificity, which might have led to higher numbers of false positives in our study. We formulated the online feedback mildly, careful not to speak of *diagnosis* or *mental health problems*, to prevent incorrect interpretation. Additionally, the relatively high number of screening instruments might have led to a high overall percentage of participants who screened positive for at least one of the screeners.

Our intervention consisted of two parts. First, the participants in the intervention group underwent online screening on impaired work functioning and impaired mental health, followed by personalised feedback on their screening results. Subsequently, they were offered a tailored offer of EMH interventions. In addition, participants with impaired work functioning received an onscreen educational leaflet on how to improve their work functioning. Two scenarios might explain our not finding an effect of the intervention: *programme failure* and *theory failure* [43]: the intervention was not carried out as intended (programme failure), or the intervention is not effective (theory failure).

The process evaluation that was carried out alongside this randomised controlled trial [44] offers some information on potential programme failure. The personalised feedback was received by all participants in the intervention group, since it appeared onscreen immediately after filling out the baseline questionnaire and was sent to the participants' e-mail address automatically. The onscreen educational leaflet on how to improve work functioning was also sent automatically to participants with impaired work functioning. However, the compliance to the subsequently offered self-help EMH interventions was low. Only 28 participants logged into an EMH intervention, and 6% (N = 9) of the positively screened participants in the intervention group started an EMH intervention to at least some extent. Regarding the second part of the intervention (the EMH interventions), program failure may therefore have occurred.

Participants offered no explanation why they did not follow an EMH intervention [44]. Three explanations are conceivable. Firstly, there is a reported trend in the literature of a low perception of need for mental health interventions. Lexis and colleagues found that 43% of employees who were identified with mild to severe depressive complaints, did not report to experience health complaints themselves [45]. Codony and colleagues found that merely a third of those who had a mental disorder in the past 12 months, had a perceived need of mental healthcare [46]. Since our study regarded a preventive setting and we chose for high sensitivity in our screening, perhaps the perceived need of our participants was insufficient to motivate them to log into and follow an EMH intervention. Secondly, some of the participants (N = 9) reported problems with logging into the interventions, due to technical problems and/or inadequate computer skills, which might have posed a problem for more participants. A third explanation is that the channelling from the personalised feedback towards the EMH interventions might not have been attractive enough to encourage participants to follow an EMH intervention.

The possibility of theory failure should also be considered. The intervention consisted of screening and personalised feedback on screening results including channelling towards EMH interventions, an onscreen educational leaflet on how to improve work functioning (if applicable), and following the EMH interventions. Most of the EMH interventions that were used in our study have been found effective to reduce symptoms of impaired mental health in previous research [14], [16], [27]–[29], supporting our hypothesis that an EMH-approach to WHS, including EMH interventions (if complied to), might be effective in improving mental health and improving work functioning. However, it should be noted that for these previous studies, most participants had actively responded to advertisements targeting people who wanted to work on their depressive symptoms or their mental fitness. Therefore, these participants actively sought help and improvement through EMH interventions. This differs considerably from our setting, as our participants took part in WHS targeting work functioning and mental health and might not have been as much aware that they would be offered EMH interventions.

However, since the intervention was not carried out as intended, we cannot conclude that the complete EMH-approach to WHS targeting work functioning and mental health of healthcare employees is ineffective. Moreover, we found that when looking at the total sample of participants, both groups improved over time, but the EMH-approach group continued to improve between three and six months after baseline while the control group slightly deteriorated in this time interval. Possibly, we were able to find a significant effect in this total sample, because the number of participants was higher in this group and the analysis was therefore better powered to find existing differences. Since the EMH interventions themselves were hardly followed, this suggests that the other elements of the complete EMH-approach – possibly increasing awareness - might have had some (delayed) effect on work functioning. However, the results in this total sample of participants are not easily interpreted, since only the personalised feedback was received by all participants in the intervention group, and the observed effect did not occur until later in time.

### Limitations

Several limitations of our study can be noted. First of all, we did not meet our required sample size for sufficient power, set at 189 participants in each group who completed participation. This increases the chance of finding non-significant p-values despite trends for differences. The data show that, regarding impaired work functioning, a higher percentage of participants in the EMH-approach group than in the control group improved to a relevant degree compared to their own baseline scores, but this difference was not statistically significant, which might have been a result of insufficient power. However, the observed effect sizes were very small, and in most cases were fairly similar between the groups.

A second limitation of our study was the fairly high and selective drop-out rate of participants, especially in the intervention group. Drop-outs had higher scores on impaired work functioning at baseline and three months follow-up than participants who did not drop out of the trial. We do not know why this occurred, since we did not assess reasons for drop-out. We received mixed reactions to the personalised but automatic feedback on screening results and the for some participants unexpected offer to follow an EMH intervention. We suppose this might have led to resistance and the higher drop-out in the intervention group. The high and selective drop-out may have introduced bias, although we have no way of knowing in which direction this possible bias occurred.

Thirdly, as discussed before, the compliance to the offered EMH interventions was low, complicating studying the effect of the complete EMH-approach to WHS.

Lastly, we studied the effects of the EMH-approach in a group of positively screened participants, regardless of what they screened positive for. Since not everyone screened positive for every impairment of complaints and the offered intervention was tailored to each individual, it might not be reasonable to expect an improvement for every impairment or complaint if examining the total group.

### Implications for practice and further research

Our study confirms that preventive actions are essential for nurses and allied health professionals, since we identified that more than 80% of participants show at least some level of impaired work functioning and/or symptoms of mental health problems.

We endeavoured to improve work functioning and mental health through online screening, personalised feedback, and a subsequent tailored offer of self-help EMH interventions. We think that targeting work functioning is an important approach, as the ultimate goal of occupational healthcare is to keep employees functioning well and as healthy as possible. However, we were unsuccessful in studying the EMH-approach, because very few participants followed an EMH intervention to at least some degree. Therefore, we recommend further research on two aspects. First, it is essential to identify the specific needs and wishes that nurses and allied health professionals have regarding their work related health and to study how they want to be supported to stay healthy and well-functioning at work. Possibly, a more comprehensive WHS including important other factors of their work, such as physical aspects (e.g. musculoskeletal complaints), would increase their interest and participation. Secondly, it should be investigated whether EMH interventions are suitable and acceptable for a WHS setting for nurses and allied health professionals, and if they would prefer some degree of contact with a healthcare provider. It is recommended to explore the possibility of “blended care”, i.e. combining an offer of an EMH intervention with several coaching sessions. Moreover, it could be useful to apply elements of persuasive design to encourage employees to follow an EMH intervention [47], [48].

## Supporting Information

Algorithm S1 **Algorithm for determining the specific choice of e-mental health interventions.**
(PDF)

Checklist S1 **CONSORT Checklist.**
(DOC)

Protocol S1 **Study protocol as published in BMC Public Health (** http://www.biomedcentral.com/1471-2458/11/290 **).**
(PDF)

Protocol S2 **Study protocol as approved by ethics committee.**
(PDF)
