# Supplementary material for: Effect of an E-mental Health Approach to Workers' Health Surveillance versus Control Group on Work Functioning of Hospital Employees: A Cluster-RCT
Source: PLoS One. 2013 Sep 12;8(9):e72546. doi: 10.1371/journal.pone.0072546 (PMC3772000; doi:10.1371/journal.pone.0072546)

Appendix 1: Algorithm for determining the specific choice of e-mental health interventions

a. *Decision rules*

- If the participant screens positive on anxiety, he or she is screened on panic disorder.
- If the participant screens positive on suicide risk, the participant is advised to seek contact with their general physician. They can also indicate that they prefer to receive an invitation for an appointment with their occupational physician. A link to the national online suicide prevention platform (113 Online) is provided, where they can seek help anonymously. No advice on how to improve their work functioning (if applicable) is given and no e-mental health interventions are offered, as we regard it most important to seek help for the suicidal thoughts.
- The offered choice of e-mental health interventions is as specific as possible for the (combination of) mental health complaint(s).
- Freedom of choice of the participant is an important aspect. Therefore, if possible, different options are given so that the participant can choose. When necessary, a priority is given by means of 'recommended' or 'optional'.
- As we do not dispose of a specific e-mental health intervention directed towards anxiety (other than panic), if the participant screens positive on only anxiety and no panic the advice is given to seek contact with the occupational physician if the complaints persist.
- The e-mental health intervention Strong at Work is not offered as an option if the mental health complaints are not related to work.
- If the participant screens positive on distress and/or work-related fatigue and 'more severe' mental health problems, the offered choice is only directed towards the 'more severe' mental health problems.
- If the participant screens positive on two or more mental health complaints (more severe than distress and/or work-related fatigue), the e-mental health intervention Psyfit is no longer given as an option because we prefer someone to follow a 'stronger' intervention.
- If the participant screens positive on two or more mental health complaints (more severe than distress and/or work-related fatigue) for which we can offer a specific e-mental health intervention (i.e. depression, panic and risky drinking behaviour), the advice is given to prioritise according to their own suffering (i.e. what do they feel they suffer from the most?).
- If the participant screens positive on three or more mental health complaints (more severe than distress and/or work-related fatigue), the advice is given to also seek contact with the occupational physician.

b. Schematic representation of the algorithm

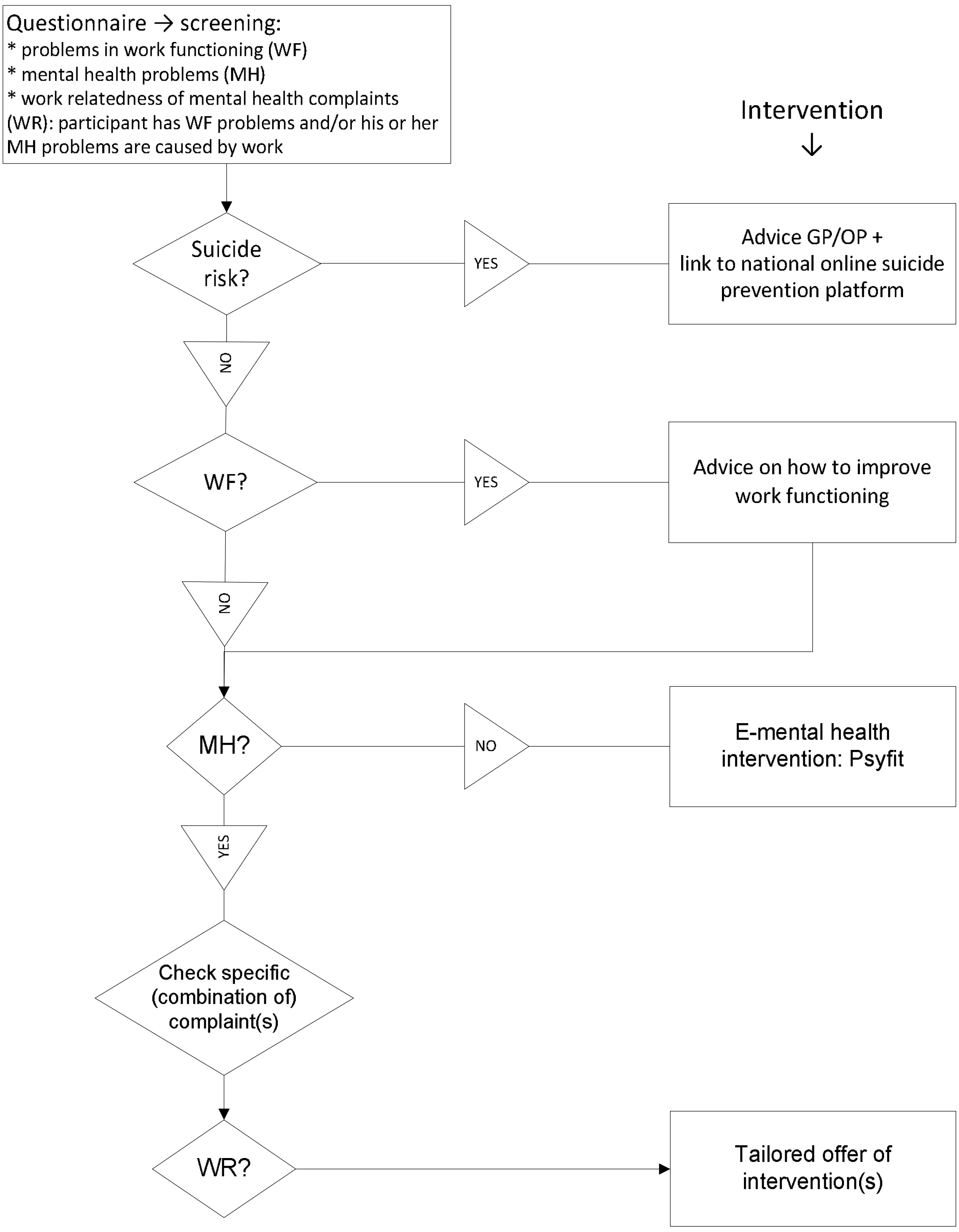

c. Example of specific algorithm for participants who screen positive for depression

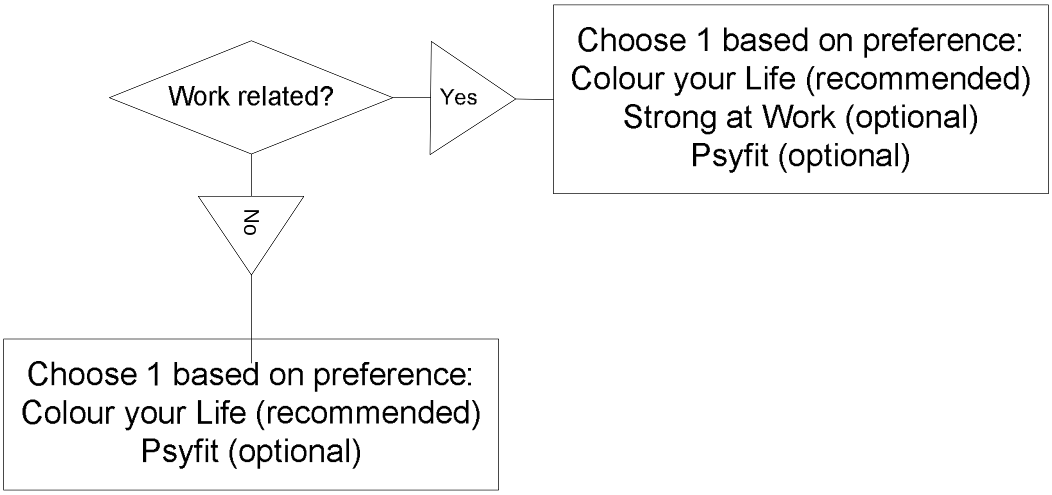

Supplement: Algorithm S1 — Algorithm for determining the specific choice of e-mental health interventions. (PDF) [file pone.0072546.s001.pdf]
