# Supplementary material for: Effect of an E-mental Health Approach to Workers' Health Surveillance versus Control Group on Work Functioning of Hospital Employees: A Cluster-RCT
Source: PLoS One. 2013 Sep 12;8(9):e72546. doi: 10.1371/journal.pone.0072546 (PMC3772000; doi:10.1371/journal.pone.0072546)
Supplement: Protocol S1 — Study protocol as published in BMC Public Health ( http://www.biomedcentral.com/1471-2458/11/290 ). (PDF) [file pone.0072546.s003.pdf]

BMC Public Health

STUDY PROTOCOL Open Access

The Mental Vitality @ Work study: design of a randomized controlled trial on the effect of a workers' health surveillance mental module for nurses and allied health professionals

Fania R Gärtner[1*], Sarah M Ketelaar[1], Odile Smeets[2], Linda Bolier[2], Eva Fischer[2], Frank JH van Dijk[1], Karen Nieuwenhuijsen[1] and Judith K Sluiter[1]

Abstract

Background: Employees in health care service are at high risk for developing mental health complaints. The effects of mental health complaints on work can have serious consequences for the quality of care provided by these workers. To help health service workers remain healthy and productive, preventive actions are necessary. A Workers' Health Surveillance (WHS) mental module may be an effective strategy to monitor and promote good (mental) health and work performance. The objective of this paper is to describe the design of a three arm cluster randomized controlled trial on the effectiveness of a WHS mental module for nurses and allied health professionals. Two strategies for this WHS mental module will be compared along with data from a control group. Additionally, the cost effectiveness of the approaches will be evaluated from a societal perspective.

Methods: The study is designed as a cluster randomized controlled trial consisting of three arms (two intervention groups, 1 control group) with randomization at ward level. The study population consists of 86 departments in one Dutch academic medical center with a total of 1731 nurses and allied health professionals. At baseline, after three months and after six months of follow-up, outcomes will be assessed by online questionnaires. In both intervention arms, participants will complete a screening to detect problems in mental health and work functioning and receive feedback on their screening results. In cases of impairments in mental health or work functioning in the first intervention arm, a consultation with an occupational physician will be offered. The second intervention arm offers a choice of self-help e-mental health interventions, which will be tailored based on each individual's mental health state and work functioning. The primary outcomes will be help-seeking behavior and work functioning. Secondary outcomes will be mental health and wellbeing. Furthermore, cost-effectiveness in both intervention arms will be assessed, and a process evaluation will be performed.

Discussion: When it is proven effective compared to a control group, a WHS mental module for nurses and allied health professionals could be implemented and used on a regular basis by occupational health services in hospitals to improve employees' mental health and work functioning.

Trial RegistrationNTR2786

* Correspondence: F.R.Gartner@amc.uva.nl
[1]Coronel Institute of Occupational Health, Academic Medical Center, University of Amsterdam, the Netherlands
Full list of author information is available at the end of the article

BioMed Central

© 2011 Gärtner et al; licensee BioMed Central Ltd. This is an Open Access article distributed under the terms of the Creative Commons Attribution License (http://creativecommons.org/licenses/by/2.0), which permits unrestricted use, distribution, and reproduction in any medium, provided the original work is properly cited.

Background

Common mental disorders (CMDs) can have negative effects on work as they can impair work functioning and increase sickness absence [1-5]. In some occupations, the impairments in work functioning can have serious consequences, such as injuries to workers. One occupation in which this vulnerability is highly present is nursing. Nurses, the largest occupational group in healthcare, are at higher risk of developing mental health problems compared to workers outside of the health care sector and compared to other (health) service workers [6]. The relative risk for depression is high for nurses, RR = 3.5, 95% CI (1.3, 9.6), compared to other human service workers and other healthcare workers [6]. This high risk might partly be explained by the very nature of the work, with work environment characteristics that are known to promote the occurrence of mental health complaints, such as high job demands, low job control and low social support [7,8]. Furthermore, in the health care sector, impairments in work functioning can have serious effects not only for the nurses but also for patients and their safety as a recent literature review showed [8,9].

In the Netherlands, the occupational health care that is provided for employees with mental health problems can be considered effective. Care according to the guidelines for occupational physicians' (OP) treatment of workers with mental health problems has been proven to improve mental health and to enhance return to work for sick-listed employees [10,11]. However, the health service for OPs is often not used by workers until they are sick-listed. Late or no help-seeking for mental health complaints is a well-known problem inside and outside of the occupational health service [12,13]. Preventive actions are needed to provide timely help before work functioning is reduced to the extent that workers cause serious incidents or must call in sick. Early identification of health complaints and risks in work functioning to provide timely help is a first step in the prevention of more serious consequences for the health and safety of the nurses and their patients. Furthermore, preventive actions can improve the wellbeing of employees in the health care sector. Wellbeing can have positive effects on the engagement and productivity of employees. With the age of the caring workforce increasing, the importance of sustainable labor participation by senior employees is increasing. Therefore, it is of utmost importance to keep the caring work force engaged and mentally fit so they can continue to meet the high mental demands of modern-day work.

A Workers' Health Surveillance (WHS) mental module may be a successful preventive strategy for CMDs and impairments in work functioning in the health care sector. Within the occupational health care setting, WHS is a well-developed strategy for preventive actions [14,15]. WHS aims to detect negative health effects of work in an early stage to enable timely interventions [15]. Although the use and application of WHS is rising for various occupations and health effects, little is known about WHS targeting mental health effects. In a recent literature review by Plat et al. [16] on WHS in military and emergency service personnel, three studies included psychological health aspects, one in police personnel [17], one in rescue and recovery workers [18] and one in soldiers [19]. WHS for mental health effects in nurses has not yet been scientifically evaluated. Therefore, the aim of this study is to test the effectiveness of a job-specific WHS mental module for nurses and allied health professionals.

Although the International Labour Organization has formulated recommendations for the use of WHS, the design differs between countries. In the Netherlands, a policy guideline on how to conduct WHS exists [20]. This guideline does not prescribe any specific interventions, but includes principles and leading criteria such as the statement that screening for health problems should only be conducted if effective interventions for that health problem are available. Furthermore, one of the core aims of the guideline is the monitoring and improvement of both the health and functioning of workers. Therefore, our job-specific WHS mental module includes screening for early signals of mental health complaints and for impairments in work functioning. For the detection of impaired mental health, several validated instruments exist that are suited for the working population. However, until recently, no instrument for detecting impaired work functioning in healthcare workers related to mental health problems was available. Such an instrument has now been developed to be used in the hospital environment, the Nurses Work Functioning Questionnaire (NWFQ) (Gärtner, Nieuwenhuijsen, van Dijk, Sluiter, unpublished). The NWFQ was designed based on literature studies and focus group investigations with the workers' supervisors, human resource managers and occupational health professionals. The NWFQ has a high content validity, and its seven subscales show good or acceptable internal consistency.

For the interventions that follow the screening, two different strategies were developed. The first strategy is a consultation offered by the OP following a protocol for care for workers with mental health complaints, as developed for this study. The second strategy is a choice of self-help e-mental health interventions that is offered to all workers - those with and without complaints. The choice is tailored to the individual screening results.

The objective of the Mental Vitality @ Work study is to study the effectiveness of two strategies for the WHS mental module in one cluster randomized controlled trial design with three arms. Substudy 1 aims to test the effectiveness of screening for problems in mental health and work functioning plus advice on appropriate interventions by an OP compared to a control group. It will study the effects on adequate help-seeking behavior, work functioning and mental health. We hypothesize that employees who receive the WHS mental module with screening plus an invitation for OP-care will show more adequate help-seeking behavior than employees in the control group. Furthermore, we hypothesize that work functioning and mental health problems will improve in employees who receive the WHS mental module with screening plus invitation for OP-care compared to employees in the control group.

Substudy 2 aims to compare the OP-care strategy with a second strategy, including the same screening of problems in mental health and work functioning as in the OP-care strategy plus a stepped care e-mental health approach. Substudy 2 will compare the effects of both strategies on work functioning and mental health. We hypothesize that both WHS mental module strategies are equivalent in their effectiveness on work functioning, mental health and wellbeing compared to the control group. An economic evaluation of the WHS mental module will be conducted alongside the randomized controlled trial. Cost-effectiveness of the WHS mental module will be assessed from a societal perspective. The employer's perspective will be considered in a cost-benefit analysis. Regarding cost effectiveness, we hypothesize that the WHS mental module with E-mental health interventions is more cost effective than the WHS mental module with OP-care.

Methods/design

In the following description of the design of the Mental Vitality @ Work study we follow the CONSORT statement, which aims to improve the quality of reporting randomized controlled trials (RCT) [21,22].

Study design

A cluster randomized controlled trial with three parallel arms will be performed in order to evaluate the effectiveness of two strategies for a WHS mental module for nurses and allied health professionals: the OP-care strategy and the E-mental health strategy. The study combines two separately funded substudies. Substudy 1 will compare the control arm with the OP-care arm - a screening on mental health complaints and impaired work functioning followed by a consultation with an OP and appropriate interventions if necessary. Substudy 1 will test the effect on help-seeking behavior, work functioning and mental health of employees with problems in mental health and/or work functioning.

Substudy 2 will compare the E-mental health arm - a screening on mental health complaints and impaired work functioning followed by a tailored choice of self-help e-mental health interventions - with the control arm and with the OP-care arm. Additionally, a subgroup analysis of the healthy participants comparing the E-mental health arm with the OP-care arm will be conducted. Substudy 2 will test the effect of the interventions on work functioning and mental health.

Participants will be followed for six months. Two follow-up measures will be conducted, one at three months and one at six months. The Medical Ethics Committee of the Academic Medical Center in Amsterdam (AMC) gave approval for the study. Figure 1 presents an overview of the study design. Below, differences between Substudy 1 and Substudy 2 are described. Otherwise, the information is equal for both parts.

Setting

The study will be performed in one Dutch academic medical center, a hospital with 7000 employees and 1102 beds. The organization has its own in-company occupational health service. Each of the different divisions in the medical center has one OP assigned for its occupational health care. In the Dutch occupational health care system, OPs fulfill preventive tasks, have the duty of detecting occupational diseases and provide (return-to-work) counseling for sick-listed employees. In the Netherlands, if they are reported sick, workers are required to visit their OP for independent judgment of sick leave and for return-to-work guidance. Furthermore, all workers can make use of the free accessible consulting hour for employees with questions about work and health [23]. According to the in-company occupational health service, usage of the free accessible consultations by employees is limited.

Study population

The research population includes all nurses, including surgical nurses and anesthetic nurses, and allied health professionals working at one Dutch academic medical center. In total, 1731 nurses and allied health professionals work in the medical center in 86 different wards, including outpatient wards. Employees who are sick-listed at the start of the study and expected to be on sick leave for more than two weeks are excluded from the study, as they will in any case consult an OP.

Recruitment of participants

For a successful intervention study in a work setting, all layers of the organization must fully support the study. As we received endorsement from the board of

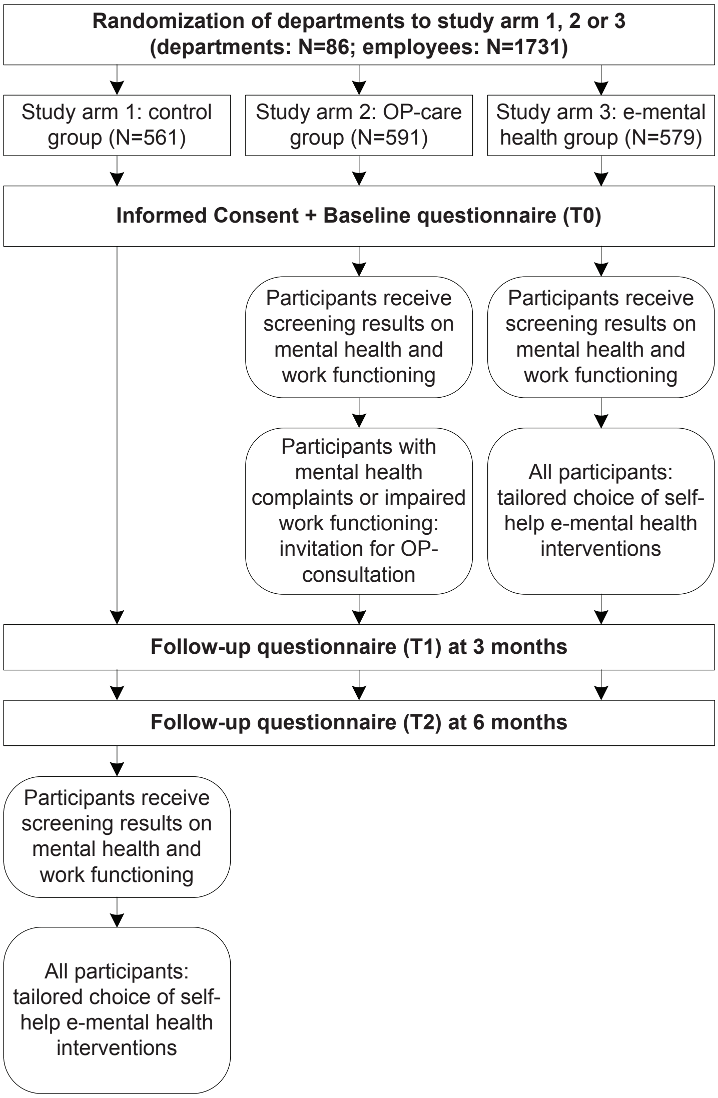

Figure 1 Study design.

directors, the nurse directors and the workers' counsel to perform the study, the likelihood for the departments to accept participation is high and all departments are expected to participate. During the enrollment period, information will be given on the hospital intranet, posters will be put up in the hospital building and flyers will be given out to promote the study.

Prior to the recruitment of individual employees, all wards will be informed about the Mental Vitality @ Work study by personal letters to the nurse directors and head nurses and the managers of allied health professionals. Subsequently, the individual workers will be informed by a letter to their home address with detailed information about the study procedure and about the safety and privacy of the individuals. Ten days later, an e-mail will be sent to the employees' work-email account, encompassing study information, a link to the online questionnaire and a personal log-in name and password. Agreeing with the informed consent, which is shown online prior to the questionnaire, is a prerequisite for starting the questionnaire. It will be possible for participants to log in on the website at any time and from any computer. It is also possible to log out at any point during the survey and to continue after logging in again. In the four weeks after the invitation for the study, three reminders will be sent to employees who have not yet started or completed the questionnaire.

Interventions

OP-care

The first WHS mental module strategy encompasses an online screening for mental health complaints and work

functioning problems plus an optional consultation with an OP for employees with mental health problems and/or work functioning problems. Directly after filling out the screening questionnaire, participants will automatically receive digital feedback on the screening results. Participants who score above a cut-off point for either the mental health complaints, work functioning impairments or both will receive an invitation from the in-company occupational health service for a face-to-face consultation with their OP scheduled within two weeks after filling out the screening questionnaire at baseline. The consultation is voluntary and supervisors of employees will not be informed about the invitation or the content of the consultation with the OP.

In order to structure the consultation of the OP, a seven-step protocol will be applied. The seven steps are: 1) discussing expectations; 2) discussing screening results and characteristics of mental health/work functioning complaints; 3) discussing possible causes in the private, work and medical situation and consequences for performing the work; 4) giving a diagnosis and offering a rationale; 5) giving advice for reduction of health complaints and for the improvement of work functioning and the prevention of incidents at work and discussing communication with the supervisor; 6) discussing a possible follow-up trajectory or referral to other care givers; and 7) summarizing the consultation.

This protocol closely follows the care as usual of the OPs. It was developed by means of interviews with the five participating OPs and based on the evidence-based guideline for OP's treatment of workers with mental health problems, which was developed by the Dutch Society of Occupational Medicine [11,24]. The main difference with the care as usual is the focus on identifying impairments in work functioning and giving advice on the improvement of work functioning and the prevention of consequences of impaired work functioning. All participating OPs were trained in using the protocol for the consultations.

E-mental health

The second WHS mental module strategy encompasses an online screening for mental health complaints and work functioning problems plus tailored advice on self-help e-mental health interventions. In this strategy, after filling out the screening questionnaire, feedback on results will be provided digitally. Workers with impaired work functioning will be digitally offered advice on how to improve their work functioning. Furthermore, an electronic health intervention trajectory will be offered to each participant to improve mental health and wellbeing. The trajectories offered for improvement of mental health will be tailored to the needs of the worker as assessed by the screening. The e-mental health interventions that can be offered are:

- Psyfit: aimed at the promotion of wellbeing. It is suitable for everyone, including healthy participants [25]. The effectiveness of Psyfit is currently being examined (Bolier, Bohlmeijer, Haverman, Boon, Kramer, Riper, unpublished).
- Strong at work (Sterk op je werk): aimed at gaining insight into work stress and at learning skills to cope with work stress.
- Colour your life (Kleur je leven): aimed at tackling depressive symptoms. Research has shown Colour your life to be (cost) effective [26-31].
- Don't Panic Online (Geen Paniek Online): aimed at reducing panic symptoms for sub-clinical and mild cases of Panic Disorder. This intervention is based on Don't Panic (*Geen Paniek*), a face-to-face group course for sub-clinical and mild panic symptoms. Don't Panic has proven to be cost-effective [32-35]. The effectiveness of this online intervention is currently being studied [36].
- Drinking less (Minder drinken): aimed at reducing risky alcohol drinking behavior and shown to be effective [37,38].

The e-mental health interventions are self-help programs on the internet aimed at reducing specific mental health complaints or enhancing wellbeing. The programs are mainly based on cognitive behavioral therapy principles and combine a variety of aspects, for instance, advice, weekly assignments, the option of keeping a diary and a forum to get in contact with others who have similar complaints. The self-help e-mental health interventions were developed by the Trimbos-institute. E-mental health programs have been shown to be effective at improving impaired mental health [28,29,37-39] and at enhancing wellbeing [40,41].

Control group

In the control arm, participants will fill out the baseline questionnaire; however, results of the screening-questionnaires will not be reported back to participants, and no further interventions will be advised at baseline. As compensation, participants in the control arm will receive their personal screening results together with a tailored choice for a self-help e-mental health intervention six months after baseline, which is identical to the intervention in the E-mental health arm at baseline.

For ethical reasons, a suicide-risk protocol is implemented in all study arms. Participants identified as being at high risk of suicide will receive immediate feedback on their self-reported suicide risk. They will be advised to seek help instantly, and they are asked to choose between

either contacting their general practitioner or receiving an invitation for an urgent consultation with their OP.

Co-interventions

To our knowledge, no co-interventions on the organization or ward level aimed at mental health or work functioning improvement will be taking place in the medical center at the time of this study.

Measures

Screening instruments used at baseline

Impaired work functioning Impaired work functioning will be measured using the job-specific Nurses Work Functioning Questionnaire (NWFQ) (Gärtner, Nieuwenhuijsen, van Dijk, Sluiter, unpublished). The NWFQ aims to measure impaired work functioning due to CMDs in nurses and allied health professionals. This 50-item self-report questionnaire consists of seven subscales: 1) *cognitive aspects of task execution and general incidents*; 2) *impaired decision making*; 3) *causing incidents at work* (not applicable for allied health professionals); 4) *avoidance behavior*; 5) *conflicts and annoyances with colleagues*; 6) *impaired contact with patients and their family*; and 7) *lack of energy and motivation*. Cronbach's alphas vary between 0.70 and 0.94. Response formats vary between 5-category and 7-category scales; however, the number of categories is the same for all items of one subscale. The content of the response scales varies between Likert-type scales (0 = *totally disagree* to 6 = *totally agree*; 0 = *disagree* to 4 = *agree*; 0 = *no difficulty* to 6 = *great difficulty*), relative frequency categories (0 = *almost never* to 6 = *almost always*; *0 = almost never* to 4 = *almost always*), and absolute frequency categories (0 = *not once* to 6 = *in general more than once a day*). Sum scores of the subscales range from 0-100. As yet, no validated cut-off scores exist for this fairly new questionnaire. Based on prior data of the study population (Gärtner, Nieuwenhuijsen, van Dijk, Sluiter, unpublished), cut-off values were calculated according to the following principle. Sumscores on the different subscales can lead to three categories: green, orange, and red. Therefore, two cut-off values are set, at the 67th percentile and at the 75th percentile of participants with mental health complaints. In two of the 7 subscales (subscale 2 and 4) the cut-off values for orange and red were identical due to little variation; in this case, cut-off values were set at the 75th and 85th percentiles of participants with mental health complaints. For the total NWFQ, a red score on one subscale or three or more orange scores will lead to case identification of impaired work functioning. In the prior dataset, this resulted in 31% of the total sample.

Distress Distress will be measured with the distress subscale of the Four-Dimensional Symptoms Questionnaire (4DSQ) [42,43]. The 16-item questionnaire uses a 5-point response scale (0 = *no*, 4 = *very often*) and has a Cronbach's alpha of 0.90 [43]. For case identification, a cut-off point of ≥11 will be applied [44].

Need for recovery Early symptoms of work-related fatigue will be measured using the Need for recovery subscale of the Dutch Experience and Evaluation of Work (Dutch: VBBA) questionnaire [45]. The 11-item questionnaire with dichotomous response categories (*yes, no*) has a Cronbach's alpha of 0.86 [46-48]. A cut-off point of ≥6 will applied. This gives a sensitivity of 0.72 and a specificity of 0.79 [49].

Alcohol use To measure risky drinking behavior, the 3-item AUDIT-C will be used. The three items ask for frequency of specific drinking behavior, varying in formulations for the items [50]. Two items have a 5-point response scale, and 1 item has a 6-point response scale. The cut-off score is ≥5 for men with a sensitivity of 90.9 and specificity of 68.4 and ≥4 for women with a sensitivity of 92.4 and specificity of 74.3 [51].

Depression and Anxiety Depression and anxiety will both be measured with the corresponding subscales of the Brief Symptom Inventory (BSI) [52]. Each subscale has six items with a 5-point response scale (0 = *not at all*, 4 = *extremely*). Cronbach's alphas are 0.87 for both scales [52]. For both subscales, mean scores of ≥0.42 are used for case identification, with a sensitivity of 0.86 and a specificity of 0.66 for depression and a sensitivity of 0.83 and a specificity of 0.62 for anxiety [53].

Suicide risk One item of the BSI depression subscale asks for suicidal thoughts. An answer on this item in one of the upper two response categories (*rather a lot* or *extremely*) will identify a person as being at high risk for suicide.

Panic disorder The panic module of the Patient Health Questionnaire (PHQ-15) will be used for the assessment of panic disorders; however, it will only be used in participants identified as having anxiety complaints [54]. The 15 items have dichotomous answering categories (*yes, no*) and a Cronbach's alpha of 0.80 [55]. For case identification, we use the following procedure: two answers affirmative on the first four items plus four symptoms affirmative on the following 11 items. This identification procedure has a sensitivity of 0.91 and a specificity of 0.88 [56].

Post traumatic stress disorder Post traumatic stress disorder is measured by the Schok Verwerkings Lijst (SVL) [57], a Dutch translation of the Impact of Event Scale [58]. The 15 items can be answered on a 4-point response scale (0 = *not at all*, 3 = *often*). Van der Ploeg et al. [59] found a Cronbach's alpha of 0.94 in a work-related sample. A cut-off point of ≥26 is applied [60].

Work relatedness of mental health complaints Work relatedness of mental health complaints is measured by

one item: "Do you think that your work has negative consequences for your mental health?" This self-formulated item has a dichotomous response scale (*yes, no*).

At T2, the same screeners will be used in the control arm.

Primary outcomes measured at baseline, three month follow-up and six month follow-up

Substudy 1 The primary study parameter of the comparison between the OP-care arm and the control arm is help-seeking behavior. It regards formal help sources that the subject has used during the past three months. In the operationalization of formal help sources, 11 help sources are presented (i.e., psychologist, psychiatrist, general practitioner, OP, physiotherapist, supervisor, coach, in-company social worker, social worker, religious counselor, alternative therapeutic treatments). The list of help sources is developed in analogy with earlier studies on help-seeking behavior [61-64].

The outcome measure help-seeking behavior is dichotomized into 'did seek formal help' for participants who had made use of any of the 11 caregivers and 'did not seek formal help' if none of the 11 caregivers were visited.

Substudy 2 The primary outcome measure of the comparison of the E-mental health arm with the OP-care and the control arm is work functioning, operationalized as job-specific impairments in work functioning. It will be measured using a total score of the Nurses Work Functioning Questionnaire (NWFQ).

Secondary outcomes measured at baseline, three month follow-up and six month follow-up

Secondary outcomes of both Substudy 1 and Substudy 2 are mental health complaints and absenteeism. The secondary outcomes that are only measured for Substudy 1 are work functioning and additional help-seeking information (intention to seek help, work as content of the consultation, frequency of visits, and informal help-seeking behavior). The secondary outcomes that are only measured for Substudy 2 are work ability, turnover intention, wellbeing, and work productivity.

Mental health complaints Mental health complaints are operationalized as the six mental health complaints screened for (i.e., distress, need for recovery, alcohol use, depression, anxiety and posttraumatic stress disorder). These are measured as described above.

Absenteeism Three items from the Productivity and Disease Questionnaire (PRODISQ) Module C are used to measure absenteeism from work. Absenteeism is operationalized as number of days on sick leave during the last three months and number of periods of sick leave during the last three months [65].

Work functioning Work functioning will be measured by the NWFQ as described above.

Additional information on help-seeking behavior Additional information concerning help-seeking behavior will be used as a secondary outcome measure, which includes 1) intention to seek help, assessed for the 11 formal help sources, 2) work as content of the consultation of various caregivers, 3) frequency of visits to the caregivers and 4) informal help-seeking behavior towards family or friends.

Work ability Work ability will be assessed with the first item of the Work Ability Index (WAI) [66]. This item concerns the evaluation of current work ability compared to their lifetime best on an 11 point scale (0 = *completely unable to work*, 10 = *work ability at its best*).

Turnover intention Turnover intention will be assessed by one item on plans to seek for a job outside of the present organization during the next year. The item can be answered on a dichotomous response scale (*yes, no*).

Wellbeing Wellbeing is measured with three questionnaires measuring different concepts.

The Mental Health Continuum-Short Form (MHC-SF) is a 14-item self-report questionnaire on wellbeing in the categories 'languishing', 'moderate' and 'flourishing' [67]. The MHC-SF measures hedonistic wellbeing as well as psychological and social wellbeing. Participants rate the items on a 6-point scale (0 = *never*, 5 = *every day*). The MHC-SF has shown good internal consistency (> 0.80) and discriminant validity [68,69].

The WHO-5 wellbeing scale contains five positively formulated items on mental health. Participants are asked to rate the items using a 6-point scale (0 = *never*, 5 = *all of the time*). The WHO-5 has been validated in different populations with an acceptable internal consistency (Cronbach's alpha 0.84) [70].

The Utrecht Work Engagement Scale (UWES-9, short-form) measures engagement at the workplace. It is a 9-item scale, and items are scored on a 7-point rating scale (0 = *never*, 6 = *always*). Cronbach's alpha of the UWES-9 varied between 0.85 and 0.92 across 10 different countries, including the Netherlands [71].

Work productivity Three items from the Productivity and Disease Questionnaire (PRODISQ) Module E are used to measure productivity losses due to presenteeism. The three items refer to the last work day, and they assess the amount of inefficient job performance, the quality loss of the work, and, if applicable, the reason for productivity loss [65].

Independent measures at baseline

As independent measures, we assess demographic characteristics, job characteristics and psychosocial work characteristics at baseline. Demographic characteristics, gender, age (in years), civil status (five categories), and ethnic background (three categories) will be assessed with self-formulated questions. As job characteristics,

we will measure the occupation, nursing specialty (if applicable), work experience in years, work hours per week, and type of labor contract. Psychosocial work characteristics will include job demands, job control, social support at work from the supervisor, and social support at work from colleagues, which will each be measured by one self-formulated item on a visual analogue scale (VAS) (0 = *not*, 100 = *to great extent*). Additionally, one item will be added for conflicts at work with the supervisor or with colleagues. As possible prognostic factors for help-seeking behavior, we include gender [72-74], civil status [75], informal help-seeking towards family or friends [76], and previous experiences with mental health care, which is operationalized as having friends or family who were treated by a psychologist/psychiatrist at any time, or having been treated by a psychologist/psychiatrist himself/herself at any time [77].

Process indicators measured at three month follow-up

Process indicators for the feasibility evaluation of the WHS will be measured at three month follow-up (T1) and include 1) participants' compliance in both the OP-care and E-mental health arm; 2) participants' satisfaction; 3) adherence of OP to the protocol; and 4) satisfaction of OP. Participants' compliance will be assessed by objective data on response rate to the study, percentages of participants who made use of the invitation for an OP consultation or the e-mental health interventions (by track and trace); moreover, based on self-report data, the percentages of participants who followed the advice given by the OP or during the e-mental health intervention. Satisfaction of participants will be measured by self-report data on satisfaction with the provided feedback, satisfaction with the invitation for the OP consultation or the e-mental health intervention, satisfaction with the consultation by the OP or e-mental health intervention itself, and satisfaction with the advice given by the OP or given in the e-mental health intervention, including their perceived effectiveness. In the OP-care arm, protocol adherence of the OPs will be assessed by means of a checklist for each protocol step, which the OP has to fill out after each consultation with a participant of the WHS mental module. The OP's satisfaction and experiences with the WHS mental module will be assessed in a group interview after the three month follow-up.

Economic evaluation indicators at baseline, three month follow-up and six month follow-up

The cost-effectiveness of the WHS mental module will be assessed from a societal perspective. Differences in effect - job-specific impairments in work functioning - will be compared with differences in costs - costs due to intervention and health care and costs stemming from productivity losses in paid work.

The employer's perspective will be considered in a cost-benefit analysis by comparing the costs of occupational health care (including the WHS mental module) with the costs due to productivity losses in paid work. Health care utilization will be measured by the Trimbos/iMTA Cost Questionnaire for Psychiatric Illness (TiC-P) [61]. Questions on occupational healthcare utilization will be added to this questionnaire for the purpose of this study. The Productivity and Disease Questionnaire (PRODISQ) will be used to measure productivity losses due to absenteeism and presenteeism (inefficient job performance) [65].

Sample Size

Substudy 1 In a study by Isaaksson Ro [78] on help-seeking behavior in nurses with burnout, the formal help-seeking increased from 17% to 34%. Differences between the percentages of participants having sought formal help between the two study arms will be examined using a Chi-square test. For an increase of 17% with alpha = 0.05 (2-tailed) and a power of (1-beta) = 0.80, power calculation using the Nquery Advisor software results in 114 participants with mental health complaints for each of the two arms. Based on data from a prior study in this population (Gärtner, Nieuwenhuijsen, van Dijk, Sluiter, unpublished), we assume that 50% of the population will have impairments in either mental health, work functioning or both. Thus, for a comparison of workers screened positive in the control arm and the OP-care arm, 228 participants in each arm are necessary. Randomization will take place at the ward level; however, we do not expect any correlation between wards in the help-seeking behavior of their employees. Therefore, no inflation factor is used in the power calculation for Substudy 1 with the outcome measure help-seeking behavior. With an expected loss-to-follow-up of 10%, we must start the trial with N = 228/0.90 = 254 per condition at baseline.

Substudy 2 The trial is powered to detect a clinically significant effect, defined as at least 0.33 standard units when the (primary) outcome is transformed into a standardized effect size, also known as Cohen's d or the standardized mean difference. Lipsey and Wilson [79] conducted a second-order meta-analysis of psychological, educational and behavioral interventions and found that for these interventions, d = 0.33 to be corresponding with the lower bound of a medium effect size. We will conduct tests at alpha = 0.05 (2-tailed) and a power of (1-beta) = 0.80. Using Stata, it is shown that n = 145 per condition is required. For the primary outcome measure of this substudy, work functioning, no information on probable difference on ward-level exists. But as a precaution, we compensate for possible cluster effects introduced into the data because of randomization on ward-level. For cluster correction, we must multiply by

a factor 1.3, which returns 145*1.3 = 189 per condition. Assuming a loss-to-follow-up of 10%, we must start the trial with N = 189/0.90 = 210 per condition at baseline.

In sum, the required minimum number of participants is 254 for the control arm, 254 for the intervention arm 2 (according to calculations for part 1) and 210 for the intervention arm 2 (according to calculations for Sub-study 2); thus, in total, 718 participants are required for all three arms. We expect a response rate of about 45%; thus, 1596 employees must be invited to recruit the required 718 participants. As we will include 1731 employees, the source population is large enough to meet the needed sample size.

Randomization and blinding

In this controlled trial, cluster randomization will be performed at the ward level. The argumentation for cluster randomization is two-fold. First, it prevents contamination effects between participants working in the same department. Second, it is in accordance with the practice of WHS, which is usually conducted for all workers in a department. The randomization procedure will take place before the inclusion of the individual participants. In the randomization, we will stratify for ward size. Randomization will be performed using block randomization with three departments in each block. To guarantee concealment of allocation, the randomization will be performed by one researcher (KN) who is not involved in the practical recruitment of participating employers, using the computer software program Nquery Advisor.

Researchers, managers of participating departments and OPs will not be blinded for the group allocation. However, as we have a prerandomization procedure with incomplete-double-consent design without mentioning the use of a reference group in the experimental groups and vice versa [80], the head (nurses) of wards and the individual employees will receive only information that is applicable to the study-arm of their wards.

Statistical analyses

The baseline data and data of the primary and secondary parameters will be presented using descriptive statistics. The effectiveness of the intervention on the primary and secondary outcome measures will be analyzed on the employee level following the intention-to-treat-principle.

Effect evaluation To study the effect on dichotomous outcome measures Chi-square tests will be used; thus, to test differences in proportions of subjects who score positive on the outcome measure between the study arms for each time of measurement. Change of proportion of employees in outcomes at follow-up (T1 and T2) will be analyzed using Generalized Estimated Equations (GEE), with wards and participants as cluster variables (where appropriate) and study-arm, time and their interaction (study-arm × time) as co-variates under the assumptions of an exchangeable working correlation matrix. Effects of demographic characteristics and prognostic factors on dichotomous outcomes measures will also be analyzed using Generalized Estimated Equations (GEE).

Effects of continuous outcome measures will be analyzed using multiple regression analysis. A multilevel analysis of variance will be conducted (GLM mixed models, repeated measurements), with ward as the primary hierarchical level and participants as the secondary hierarchical level (where appropriate). Effects of demographic characteristics and prognostic factors on continuous outcome measures will also be analyzed using multiple regression analysis.

Cost-effectiveness evaluation For the cost-effectiveness evaluation, the incremental cost-effectiveness ratio (ICER) will be calculated by comparing the differences in costs of health care utilization and productivity losses for each WHS strategy with the difference in effect on job-specific work impairments of both strategies. The index year for health care costs will be 2011. Productivity losses will be assessed using the human capital approach. Analyses will include cost-effectiveness planes and acceptability curves. Ancillary analyses (i.e., incremental net-benefit regression analysis) will identify subgroups of workers (e.g., participants with and without impaired work functioning or mental health) who derive particular benefit from the intervention.

In the cost-benefit analysis, the employer's perspective will be considered by comparing the costs of offering the WHS modules with the costs of productivity losses due to sickness absence (absenteeism) and working less efficiently while at work (presenteeism) for both WHS strategies separately.

Process evaluation Participant compliance and participant satisfaction as well as adherence of OP will be presented in proportions. Satisfaction of OPs will be assessed in terms of strengths and suggestions for improvement.

Ethical considerations

There are no risks associated with participating in the Mental Vitality @ Work study. Confidentiality is guaranteed during the whole study for the employees of all study arms, as no information about the screening or the interventions will be provided to others, such as supervisors. Furthermore, the study participants of all study-arms retain unrestricted access to care as usual if requested. Employees and their supervisors are still free to call in any occupational health care in the medical center if they wish to do so.

Discussion

The health care service is a sector with special risks for the development of mental health complaints. In turn,

in this sector, impaired mental health can have serious consequences for the workers and their patients. A WHS mental module might be an effective preventive action to promote and monitor good (mental) health and work performance in the aging workforce. The aim of the Mental Vitality @ Work study is to test the effectiveness of two strategies for a WHS mental module for nurses and allied health professionals. This paper describes the protocol for a three-arm RCT in which the effectiveness of the two strategies for a WHS mental module will be evaluated. First, the effect on help-seeking behavior for the OP-care arm compared to a control arm and, second, a comparison of the effect on work functioning for the E-mental health arm with the OP-care arm and the control arm. Additionally, an economic evaluation of both procedures will be evaluated from both a societal and employer perspective.

WHS is a well-developed strategic concept to protect workers against health risks and to monitor and enhance their work functioning. Mental modules for WHS have been developed in some sectors, e.g., the police sector [17]. In these studies, the identification of workers in need of health care intervention was solely based on the mental health status. The innovative aspect of our approach is that, in addition to screening for mental health problems, a screening for work functioning problems is carried out. The identification of work functioning problems in workers with mental health complaints yields input for the kind of intervention needed to enhance work functioning and to prevent more serious consequences such as incidents at work. In line with this, our approach differs from other mental health screenings in the work setting, because we will test the effectiveness of the WHS mental module both at enhancing work functioning and at improving mental health.

Another innovative aspect of our study is the included e-mental health interventions. Although the effects of e-mental health interventions on mental health outcomes appear promising, applying them in the context of WHS in a specific working population is a new approach. An advantage of this context is that the e-mental health intervention can be tailored to the mental health outcome of the screening that precedes the offered interventions.

Methodological considerations

One strength of our RCT-design is the cluster randomization with pre-randomization. Applying a WHS procedure to a ward as a whole is not only in line with WHS in common occupational health service practice, but it also reduces contamination of employees. The pre-randomization approach allows blinding of participants for information of the other study arms. Still, contamination effects due to communication and occasional switching between wards cannot be ruled out completely, as the study is conducted in one organization.

One methodological issue to be considered regards our choice for not applying an inflation factor for cluster correction in Substudy 1. This choice is based on two arguments. First, we do not expect any systematic differences between the hospital wards in differences on health seeking behavior of their individual workers, which makes cluster correction illogical. Furthermore, we do not expect any noteworthy differences between the study arms in baseline characteristics, due to the large amount of clusters (N = 86). For work functioning, the primary outcome measure of Substudy 2, the possibility of systematic differences between the wards, is more likely. It is conceivable that improvement in work functioning, e.g., decision making, is more difficult for workers of one ward than workers of another ward, due to differences in work context. Therefore, a cluster correction is applied on Substudy 2.

We expect the external validity of this study to be high, as the study is encompassed in a real-life setting. Furthermore, in the set-up of the interventions, good feasibility is allowed for by using input of (nurse) managers and the occupational health service that provides the OP-care. The protocol for the OP consultations is developed based on interviews with the OPs and follows care as usual closely.

Impact of results

The output of the Mental Vitality @ Work study will be two-fold. First, two WHS mental modules for nurses and allied health professionals will be delivered. Based on results on the effectiveness together with results on the process evaluation, a WHS mental module for nurses and allied health professionals could be implemented and used on a regular basis by occupational health services in academic medical centers. A WHS mental module can be used as a stand-alone intervention or as part of a broader WHS program. With minor modifications, the module can be adapted to the context of other healthcare organizations.

Secondly, the proposed study will yield valuable knowledge on the effectiveness and cost-effectiveness of a WHS mental module. If it is effective in terms of costs and improvement of adequate help-seeking, work functioning, and improved mental health, the procedure for a WHS mental module will possibly be used as a blueprint and contribute to the development of WHS mental modules in other sectors. It also might promote the use of WHS in the Netherlands. Results of the study will become available in 2012.

Acknowledgements and funding

Substudy 1 was supported by a grant from the Dutch Foundation Institute Gak.

Substudy 2 was supported by a grant from the Netherlands institute for health research and development (ZonMw).

List of abbreviations

CMD: Common Mental Disorders; OP: Occupational Physician; RCT: Randomized Controlled Trial; VAS: Visual Analogue Scale; WHS: Workers' Health Surveillance;

Author details

[1]Coronel Institute of Occupational Health, Academic Medical Center, University of Amsterdam, the Netherlands. [2]Innovation Center of Mental Health & Technology (I.COM), Trimbos Institute, Netherlands Institute of Mental Health and Addiction, Utrecht, the Netherlands.

Authors' contributions

FG contributed to the conception and design of the study and drafted the article. SK contributed to the conception and design of the study and provided critical revision of the article. OS contributed to the conception and design of the study and provided critical revision of the article. LB contributed to the conception and design of the study and provided critical revision of the article. EF contributed to the conception and design of the study and provided critical revision of the article. FD contributed to the conception and design of the study and provided critical revision of the article. KN contributed to the conception and design of the study and provided critical revision of the article. JS contributed to the conception and design of the study and provided critical revision of the article. KN, FD, and JS obtained funding for Substudy 1. KN, LB, and JS obtained funding for Substudy 2. JS and FD were co-principal investigators. All authors provided final approval of the version to be published.

Authors' information

[1] Academic Medical Center, University of Amsterdam, Department: Coronel Institute of Occupational Health

[2] Trimbos Institute, Netherlands Institute of Mental Health and Addiction, Innovation Center of Mental Health & Technology (I.COM)

Competing interests

The authors declare that they have no competing interests.

Received: 14 April 2011 Accepted: 10 May 2011 Published: 10 May 2011

References

1. Aronsson G, Gustafsson K, Dallner M: **Sick but yet at work. An empirical study of sickness presenteeism.** *J Epidemiol Community Health* 2000, **54**:502-509.
2. Hilton MF, Scuffham PA, Sheridan J, Cleary CM, Whiteford HA: **Mental ill-health and the differential effect of employee type on absenteeism and presenteeism.** *J Occup Environ Med* 2008, **50**:1228-1243.
3. Lerner D, Adler DA, Chang H, Berndt ER, Irish JT, Lapitsky L, Hood MY, Reed J, Rogers WH: **The clinical and occupational correlates of work productivity loss among employed patients with depression.** *J Occup Environ Med* 2004, **46**:S46-S55.
4. Lerner D, Henke RM: **What does research tell us about depression, job performance, and work productivity?** *J Occup Environ Med* 2008, **50**:401-410.
5. McKnight PE, Kashdan TB: **The importance of functional impairment to mental health outcomes: a case for reassessing our goals in depression treatment research.** *Clin Psychol Rev* 2009, **29**:243-259.
6. Wieclaw J, Agerbo E, Mortensen PB, Bonde JP: **Risk of affective and stress related disorders among employees in human service professions.** *Occup Environ Med* 2006, **63**:314-319.
7. Gershon RR, Stone PW, Zeltser M, Faucett J, MacDavitt K, Chou SS: **Organizational climate and nurse health outcomes in the United States: a systematic review.** *Ind Health* 2007, **45**:622-636.
8. Sundin L, Hochwalder J, Bildt C, Lisspers J: **The relationship between different work-related sources of social support and burnout among registered and assistant nurses in Sweden: a questionnaire survey.** *Int J Nurs Stud* 2007, **44**:758-769.
9. Gätner FR, Nieuwenhuijsen K, van Dijk FJ, Sluiter JK: **The impact of common mental disorders on the work functioning of nurses and allied health professionals: A systematic review.** *Int J Nurs Stud* 2010.
10. Nieuwenhuijsen K, Verbeek JH, de Boer AG, Blonk RW, van Dijk FJ: **Validation of performance indicators for rehabilitation of workers with mental health problems.** *Med Care* 2005, **43**:1034-1042.
11. van der Klink JJ, Blonk RW, Schene AH, van Dijk FJ: **Reducing long term sickness absence by an activating intervention in adjustment disorders: a cluster randomised controlled design.** *Occup Environ Med* 2003, **60**:429-437.
12. Coles ME, Coleman SL: **Barriers to treatment seeking for anxiety disorders: initial data on the role of mental health literacy.** *Depress Anxiety* 2010, **27**:63-71.
13. Dahlberg KM, Waern M, Runeson B: **Mental health literacy and attitudes in a Swedish community sample - investigating the role of personal experience of mental health care.** *BMC Public Health* 2008, **8**:8.
14. International Labour Organization: *Technical and ethical guidelines for workers' health surveillance* Geneva: ILO; 1998.
15. Koh D, Aw TC: **Surveillance in occupational health.** *Occup Environ Med* 2003, **60**:705-10, 633.
16. Plat MJ, Frings-Dresen MH, Sluiter JK: **A systematic review of job-specific workers' health surveillance activities for fire-fighting, ambulance, police and military personnel.** *Int Arch Occup Environ Health* 2011.
17. Winwood PC, Tuckey MR, Peters R, Dollard MF: **Identification and measurement of work-related psychological injury: piloting the psychological injury risk indicator among frontline police.** *J Occup Environ Med* 2009, **51**:1057-1065.
18. Levin SM, Herbert R, Moline JM, Todd AC, Stevenson L, Landsbergis P, Jiang S, Skloot G: **Physical health status of world trade center rescue, recovery workers, volunteers-New York City, July 2002-August.** *Morb Mortal Week Report* 2004, **53**:807-812.
19. Wright KM, Huffman AH, Adler AB, Castro CA: **Psychological screening program overview.** *Mil Med* 2002, **167**:853-861.
20. NVAB: *Leidraad Preventief Medisch Onderzoek [Guidance Workers' Health Surveillance]* Utrecht, the Netherlands: NVAB [Netherlands Society of Occupational Medicine]; 2005.
21. Campbell MK, Elbourne DR, Altman DG: **CONSORT statement: extension to cluster randomised trials.** *BMJ* 2004, **328**:702-708.
22. Schulz KF, Altman DG, Moher D: **CONSORT 2010 Statement: Updated guidelines for reporting parallel group randomised trials.** *J Clin Epidemiol* 2010, **63**:834-840.
23. Hulshof CT, Frings-Dresen MH: **International OH systems Part 2: occupational health deliveray in the Netherlands.** *Occupational Health at Work* 2010, **6**:19-23.
24. van der Klink JJ: *Richtlijn: Handelen van de bedrijfsarts bij werkenden met psychisch problemen. [Guideline: management of mental health problems of workers by occupational physicians] [Netherlands Society of Occupational Medicine]* Utrecht: NVAB; 2007.
25. Haverman M, Bohlmeijer E, Bolier L: *Psyfit, een internet interventie ter bevordering van de mentale gezondheid [Psyfit, an online intervention to promote mental health]* Utrecht: Trimbos-instituut; 2010.
26. de Graaf LE, Gerhards SA, Arntz A, Riper H, Metsemakers JF, Evers SM, Severens JL, Widdershoven G, Huibers MJ: **Clinical effectiveness of online computerised cognitive-behavioural therapy without support for depression in primary care: randomised trial.** *Br J Psychiatry* 2009, **195**:73-80.
27. de Graaf LE, Huibers MJ, Riper H, Gerhards SA, Arntz A: **Use and acceptability of unsupported online computerized cognitive behavioral therapy for depression and associations with clinical outcome.** *J Affect Disord* 2009, **116**:227-231.
28. Spek V, Nyklicek I, Smits N, Cuijpers P, Riper H, Keyzer J, Pop V: **Internet-based cognitive behavioural therapy for subthreshold depression in people over 50 years old: a randomized controlled clinical trial.** *Psychol Med* 2007, **37**:1797-1806.
29. Spek V, Cuijpers P, Nyklicek I, Riper H, Keyzer J, Pop V: **Internet-based cognitive behaviour therapy for symptoms of depression and anxiety: a meta-analysis.** *Psychol Med* 2007, **37**:319-328.

30. Spek V, Cuijpers P, Nyklicek I, Smits N, Riper H, Keyzer J, Pop V: **One-year follow-up results of a randomized controlled clinical trial on internet-based cognitive behavioural therapy for subthreshold depression in people over 50 years.** *Psychol Med* 2008, **38**:635-639.
31. Warmerdam L, van SA, Twisk J, Riper H, Cuijpers P: **Internet-based treatment for adults with depressive symptoms: randomized controlled trial.** *J Med Internet Res* 2008, **10**:e44.
32. Meulenbeek P, Willemse G, Smit F, van BA, Spinhoven P, Cuijpers P: **Early intervention in panic: randomized controlled trial and cost-effectiveness analysis.** *Trials* 2008, **9**:67.
33. Meulenbeek P, Willemse G, Smit F, Smits N, van BA, Spinhoven P, Cuijpers P: **Effects and feasibility of a preventive intervention in sub-threshold and mild panic disorder: Results of a pilot study.** *BMC Res Notes* 2009, **2**:4.
34. Meulenbeek P, Willemse G, Smit F, van BA, Spinhoven P, Cuijpers P: **Early intervention in panic: pragmatic randomised controlled trial.** *Br J Psychiatry* 2010, **196**:326-331.
35. Smit F, Willemse G, Meulenbeek P, Koopmanschap M, van BA, Spinhoven P, Cuipers P: **Preventing panic disorder: cost-effectiveness analysis alongside a pragmatic randomised trial.** *Cost Eff Resour Alloc* 2009, **7**:8.
36. van Ballegooijen W, Riper H, van SA, Kramer J, Conijn B, Cuijpers P: **The effects of an Internet based self-help course for reducing panic symptoms - Don't Panic Online: study protocol for a randomised controlled trial.** *Trials* 2011, **12**:75.
37. Kramer J, Riper H, Lemmers L, Conijn B, van SA, Smit F: **Television-supported self-help for problem drinkers: a randomized pragmatic trial.** *Addict Behav* 2009, **34**:451-457.
38. Riper H, Kramer J, Smit F, Conijn B, Schippers G, Cuijpers P: **Web-based self-help for problem drinkers: a pragmatic randomized trial.** *Addiction* 2008, **103**:218-227.
39. Riper H, Smit F, van der Zanden R, Conijn B, Kramer J, Mutsaers K: *E-mental health. High tech, high touch, high trust* Utrecht: Trimbos-instituut; 2007.
40. Mitchell J, Stanimirovic R, Klein B, Vella-Brodrick D: **A randomised controlled trial of a self-guided internet intervention promoting well-being.** *Comput Hum Behav* 2009, 749-760.
41. Seligman MEP, Steen TA, Park N, Peterson C: **Positive Psychology Progress: Empirical Validation of Interventions.** *Am Psychol* 2005, 410-421.
42. Terluin B: **De Vierdimensionele Klachtenlijst (4DKL) in de huisartspraktijk [The Four Dimensional Symptom Questionnaire (4DSQ)].** *De Psycholoog* 1998, **33**:18-24.
43. Terluin B, van Marwijk HW, Ader HJ, de Vet HC, Penninx BW, Hermens ML, van Boeijen CA, van Balkom AJ, van der Klink JJ, Stalman WA: **The Four-Dimensional Symptom Questionnaire (4DSQ): a validation study of a multidimensional self-report questionnaire to assess distress, depression, anxiety and somatization.** *BMC Psychiatry* 2006, **6**:34.
44. van Rhenen W, van Dijk FJ, Schaufeli WB, Blonk RW: **Distress or no distress, that's the question: A cutoff point for distress in a working population.** *J Occup Med Toxicol* 2008, **3**:3.
45. van Veldhoven M, Meijman TF: *Het meten van een psychosociale arbeidsbelasting met een vragenlijst: de vragenlijst beleving en beoordeling van arbeid (VBBA) [The measurement of psychosocial job demands with a questionnaire: the questionnaire on the experience and evaluation of work (QEEW)]* Amsterdam: Dutch Institute for Working Conditions; 1994.
46. de Croon EM, Sluiter JK, Frings-Dresen MH: **Psychometric properties of the Need for Recovery after work scale: test-retest reliability and sensitivity to detect change.** *Occup Environ Med* 2006, **63**:202-206.
47. Sluiter JK, de Croon EM, Meijman TF, Frings-Dresen MH: **Need for recovery from work related fatigue and its role in the development and prediction of subjective health complaints.** *Occup Environ Med* 2003, **60(Suppl 1)**:i62-i70.
48. van Veldhoven MJ, Sluiter JK: **Work-related recovery opportunities: testing scale properties and validity in relation to health.** *Int Arch Occup Environ Health* 2009, **82**:1065-1075.
49. Broersen JPJ, Fortuin RJ, Dijkstra L, van Veldhoven M, Prins J: **Monitor Arboconvenanten: kengetallen en grenswaarden. [Monitor occupational health and safety: key indicators and limits].** *TBV* 2004, **12**:100-104.
50. Bush K, Kivlahan DR, McDonell MB, Fihn SD, Bradley KA: **The AUDIT alcohol consumption questions (AUDIT-C): an effective brief screening test for problem drinking. Ambulatory Care Quality Improvement Project (ACQUIP). Alcohol Use Disorders Identification Test.** *Arch Intern Med* 1998, **158**:1789-1795.
51. Gual A, Segura L, Contel M, Heather N, Colom J: **Audit-3 and audit-4: effectiveness of two short forms of the alcohol use disorders identification test.** *Alcohol Alcohol* 2002, **37**:591-596.
52. de Beurs E: *Brief Symptom Inventory (BSI): Handleiding* Leiden: Pits Publishers; 2006.
53. de Beurs E, Zitman FG: **De Brief Symptom Inventory (BSI): De betrouwbaarheid en validiteit van een handzaam alternatief voor de SCL-90. [The Brief Symptom Inventory (BSI): The reliability and validity of a brief alternative of the SCL-90].** *Maandblad GeestelijkeVolksgezondheid* 2006, **61**:120-141.
54. Spitzer RL, Kroenke K, Williams JB: **Validation and utility of a self-report version of PRIME-MD: the PHQ primary care study. Primary Care Evaluation of Mental Disorders. Patient Health Questionnaire.** *JAMA* 1999, **282**:1737-1744.
55. Kroenke K, Spitzer RL, Williams JB: **The PHQ-15: validity of a new measure for evaluating the severity of somatic symptoms.** *Psychosom Med* 2002, **64**:258-266.
56. Lowe B, Grafe K, Zipfel S, Spitzer RL, Herrmann-Lingen C, Witte S, Herzog W: **Detecting panic disorder in medical and psychosomatic outpatients: comparative validation of the Hospital Anxiety and Depression Scale, the Patient Health Questionnaire, a screening question, and physicians' diagnosis.** *J Psychosom Res* 2003, **55**:515-519.
57. Brom D, Kleber RJ: **De Schok Verwerkings Lijst [The Impact of Event Scale].** *Nederlands Tijdschrift voor de Psychologie* 2011, **40**:164-168.
58. Horowitz M, Wilner N, Alvarez W: **Impact of Event Scale: a measure of subjective stress.** *Psychosom Med* 1979, **41**:209-218.
59. van der Ploeg E, Mooren TT, Kleber RJ, van der Velden PG, Brom D: **Construct validation of the Dutch version of the impact of event scale.** *Psychol Assess* 2004, **16**:16-26.
60. Chemtob CM, Tomas S, Law W, Cremniter D: **Postdisaster psychosocial intervention: a field study of the impact of debriefing on psychological distress.** *Am J Psychiatry* 1997, **154**:415-417.
61. Hakkaart-van Roijen L, van Straten A, Donker M: *Trimbos/iMTA questionnaire for costs associated with psychiatric illness (TIC-P)* Rotterdam: iMTA; 2002.
62. Rickwood D, Deane FP, Wilson CJ, Ciarrochi J: **Young people's help-seeking for mental health problems.** *AeJAMH (Australian e-Journal for the Advancement of Mental Health)* 2005.
63. Walters K, Buszewicz M, Weich S, King M: **Help-seeking preferences for psychological distress in primary care: effect of current mental state.** *Br J Gen Pract* 2008, **58**:694-698.
64. Wilson CJ, Deane FP, Ciarrochi J, Rickwood D: **Measuring Help-Seeking Intentions: Properties of the General Help-Seeking Questionnaire.** *Canadian Journal of Counselling* 2005, 15-28.
65. Koopmanschap MA: **PRODISQ: a modular questionnaire on productivity and disease for economic evaluation studies.** *Expert Rev Pharmacoecon Outcomes Res* 2005, **5**:23-28.
66. Tuomi K, Ilmarinen J, Jahkola A, Katajarinne L, Tulkki A: *Work Ability Index.* 2 edition. Helsinki: Finnish Institute of Occupational Health; 1998.
67. Keyes CL: **The mental health continuum: from languishing to flourishing in life.** *J Health Soc Behav* 2002, **43**:207-222.
68. Lamers SMA, Westerhof GJ, Bohlmeijer ET, ten Klooster PM, Keyes CLM: **Evaluating the psychometric properties of the Mental Health Continuum-Short Form (MHC-SF).** *J Clin Psychol* 2011, **67**:99-110.
69. Westerhof GJ, Keyes CLM: **Geestelijke gezondheid is meer dan de afwezigheid van ziekte [Mental health is more then the absence of disease].** *Maandblad Geestelijke Gezondheid* 2008, **10**:808-820.
70. Bech P, Olsen LR, Kjoller M, Rasmussen NK: **Measuring well-being rather than the absence of distress symptoms: a comparison of the SF-36 Mental Health subscale and the WHO-Five Well-Being Scale.** *Int J Methods Psychiatr Res* 2003, **12**:85-91.
71. Schaufeli WB, Bakker AB, Salanova M: **The Measurement of Work Engagement With a Short Questionnaire: A Cross-National Study.** *Educ Psychol Meas* 2006, 701-716.
72. Galdas PM, Cheater F, Marshall P: **Men and health help-seeking behaviour: literature review.** *J Adv Nurs* 2005, **49**:616-623.
73. Judd F, Komiti A, Jackson H: **How does being female assist help-seeking for mental health problems?** *Aust N Z J Psychiatry* 2008, **42**:24-29.
74. Moller-Leimkuhler AM: **Barriers to help-seeking by men: a review of sociocultural and clinical literature with particular reference to depression.** *J Affect Disord* 2002, **71**:1-9.

75. Gudmundsdottir G, Vilhjalmsson R: **Group differences in outpatient help-seeking for psychological distress: results from a national prospective study of Icelanders.** *Scand J Public Health* 2010, **38**:160-167.
76. Rudell K, Bhui K, Priebe S: **Do 'alternative' help-seeking strategies affect primary care service use? A survey of help-seeking for mental distress.** *BMC Public Health* 2008, **8**:207.
77. Schomerus G, Matschinger H, Angermeyer MC: **The stigma of psychiatric treatment and help-seeking intentions for depression.** *Eur Arch Psychiatry Clin Neurosci* 2009, **259**:298-306.
78. Isaksson Ro KE, Gude T, Tyssen R, Aasland OG: **A self-referral preventive intervention for burnout among Norwegian nurses: one-year follow-up study.** *Patient Educ Couns* 2010, **78**:191-197.
79. Lipsey MW, Wilson DB: **The efficacy of psychological, educational, and behavioral treatment. Confirmation from meta-analysis.** *Am Psychol* 1993, **48**:1181-1209.
80. Schellings R, Kessels AG, ter RG, Sturmans F, Widdershoven GA, Knottnerus JA: **Indications and requirements for the use of prerandomization.** *J Clin Epidemiol* 2009, **62**:393-399.

Pre-publication history
The pre-publication history for this paper can be accessed here:
http://www.biomedcentral.com/1471-2458/11/290/prepub

doi:10.1186/1471-2458-11-290
Cite this article as: Gärtner *et al.*: The Mental Vitality @ Work study: design of a randomized controlled trial on the effect of a workers' health surveillance mental module for nurses and allied health professionals. *BMC Public Health* 2011 **11**:290.

Submit your next manuscript to BioMed Central and take full advantage of:

- **Convenient online submission**
- **Thorough peer review**
- **No space constraints or color figure charges**
- **Immediate publication on acceptance**
- **Inclusion in PubMed, CAS, Scopus and Google Scholar**
- **Research which is freely available for redistribution**

Submit your manuscript at
www.biomedcentral.com/submit

BioMed Central
