# Supplementary material for: Effect of an E-mental Health Approach to Workers' Health Surveillance versus Control Group on Work Functioning of Hospital Employees: A Cluster-RCT
Source: PLoS One. 2013 Sep 12;8(9):e72546. doi: 10.1371/journal.pone.0072546 (PMC3772000; doi:10.1371/journal.pone.0072546)
Supplement: Protocol S2 — Study protocol as approved by ethics committee. (PDF) [file pone.0072546.s004.pdf]

English translation of first letter to Medical Ethics Committee of the Academic Medical Center

Amsterdam, July 27 2010

Dear Mrs. Donselaar,

By means of this letter, I ask the executive committee of the Medical Ethics Committee to judge whether our planned research project is a scientific research project as defined in the “Act medical-scientific research with human participants”, and thus whether a medical-ethical review by your committee is required. We assume it is not but we would like to receive more certainty.

The planned research project targets “healthy functioning” of nurses, surgical nurses, anaesthetic nurses and allied health professionals in the Academic Medical Center and combines insights and used methods from Human Resource Management and Occupational Health Service. The study aim is to research the effectiveness of a newly developed module for workers’ health surveillance.

The research project includes two substudies. The first substudy researches the effectiveness of the module in terms of help-seeking behaviour (formal and informal help sources such as occupational physician, psychologists, coaches, supervisor and family). The second substudy researches the effectiveness of the module in terms of influence on work functioning and mental health.

The research questions will be researched using a randomised controlled trial with three study arms and cluster randomisation on ward level. Employees will be invited by e-mail to participate in one of the three arms, based on the allocation of their ward. After an individual informed consent, they will be asked to:

- In arm 1 (control group): fill out a questionnaire about work functioning and mental health.
- In arm 2: (intervention group 1): fill out a questionnaire, after which they will receive their personal results digitally, as well as the advice to contact the occupational physician in the open consultation hour. The occupational physician will advise the employee, using the regular interventions that are usual within the preventive consultation.
- In arm 3 (intervention group 2): fill out a questionnaire, after which they will receive their personal results digitally. Subsequently, the employees will be advised to follow an online intervention, based on their personal results. These interventions are existing interventions developed by the Trimbos Institute (Netherlands Institute of Mental Health and Addiction) which have been placed in one portal for this research project. They are all self-help intervention for topics such as coping with stress at work (“Strong at work”), decreasing feelings of depression (“Colour your Life”), and enhancing mental health (“Psyfit”). This last intervention will be offered to all employees in this study arm.

The effect assessments consist of online questionnaires after three and six months. The follow-up assessments include questionnaires on help-seeking behaviour, work functioning, mental health complaints, and wellbeing.

I hope to have informed you sufficiently to pass judgment.

Yours sincerely,

Dr. Karen Nieuwenhuijsen (project leader) and Dr. Judith K. Sluiter (Principal Investigator).
Coronel Institute of Occupational Health

English translation of response from Medical Ethics Committee of the Academic Medical Center to our first letter

Amsterdam, September 7 2010

Dear Mrs. Nieuwenhuijsen,

Your letter (dd July 27 2010) concerning the research project “Healthy functioning of nurses, surgical nurses, anaesthetic nurses and allied health professionals” has been discussed in the meeting of the executive committee of the Medical Ethics Committee on last September 2.

Considering that this research project will be carried out with employees, the executive committee would like to be informed whether the study participants can freely decide on their participation to this study. We request you to send us your response in four copies for discussion in the next meeting of the executive committee.

Yours sincerely, on behalf of the Medical Ethics Committee,

Mrs. dr. Y.E. Donselaar
Executive secretary

English translation of second letter to the Medical Ethics Committee of the Academic Medical Center (response to their request)

September 17 2010

Dear Mrs. Donselaar,

In response to your letter dd September 7 2010 concerning the research project "Healthy functioning of nurses, surgical nurses, anaesthetic nurses and allied health professionals", we inform the executive committee that our study participants are free to decide on their participation to this study. Eligible participants will be informed in advance about this freedom of choice. Supervisors will not be informed about the results of individual employees or about whether or not individual employees participated to the study.

We hope to have informed the executive committee sufficiently to pass judgment.

Yours sincerely,

Dr. Karen Nieuwenhuijsen (project leader) and Dr. Judith K. Sluiter (Principal Investigator).
Coronel Institute of Occupational Health

English translation of the judgment of the Medical Ethics Committee of the Academic Medical Center

Amsterdam, September 24 2010
Regarding: research project healthy functioning of nurses, surgical nurses, anaesthetic nurses and allied health professionals

Dear Mrs. Nieuwenhuijsen,

Your response dd past September 17 to our letter dd September 7 2010 has been discussed in the meeting of the executive committee of the Medical Ethics Committee on last September 23.

We willingly inform you that the abovementioned project in the judgment of the executive committee does not fall within the scope of the "Act medical-scientific research with human participants" since the burden on the study participants only concerns the filling out of questionnaires in combination with following up on advice for help to their own wishes through the occupational physician or through self-help interventions. Your response implies that the study participants are free to decide on their participation. Formal judgment by our committee is therefore not necessary.

Yours sincerely, on behalf of the Medical Ethics Committee,

Mrs. dr. Y.E. Donselaar
Executive secretary
